# Calculation of ultimate bearing capacity and analysis of bearing characteristics for pile group foundation to underlying offset cave

**DOI:** 10.1371/journal.pone.0344045

**Published:** 2026-03-03

**Authors:** Yan Zhao, Jiaxing Tao, Pu Li, Lujun Yin, Zhuang Zhang, Haiqin Yan, Wenzhe Zhang, Shuqi Ma, Jinglei Liu, Hailong Wang

**Affiliations:** 1 Hebei University of Architecture, Zhangjiakou, Hebei, China; 2 Zhongji Jiankan Group Co., Ltd., Shijiazhuang, Hebei, China; 3 Key Laboratory of Civil Engineering Diagnosis, Reconstruction and Disaster Resistance of Hebei Province, Zhangjiakou, Hebei, China; 4 Hebei Province Green Building Collaborative Innovation Center, Zhangjiakou, Hebei, China; 5 Shijiazhuang Tiedao University, Shijiazhuang, Hebei, China; China Construction Fourth Engineering Division Corp. Ltd, CHINA

## Abstract

Based on the pile group effect and the principle of limit analysis, a equation is derived for calculating the ultimate bearing capacity of pile group foundations under the condition of offset karst caves beneath. Relying on the pile foundation project in the Guangzhou Baiyun District Science, this study conducts an analysis of the ultimate bearing capacity and bearing characteristics of pile group foundations under karst terrain conditions. The results indicate that an increase in the offset distance of the underlying karst cave leads to a higher ultimate bearing capacity of the pile group foundation. The results of the comparative analysis demonstrate that the proposed method for calculating the ultimate bearing capacity of pile groups is reliable. This method is suitable for evaluating the ultimate bearing capacity and performing safety checks of pile group foundations under the condition of offset karst caves.

## 1 Introduction

Karst area [1] is characterized by a range of unique geological formations created through the dissolution of soluble rocks by water. The complex and heterogeneous geological structure of karst areas poses significant challenges to engineering construction and introduces unpredictable safety hazards [2,3]. Consequently, safety analysis is a critical aspect of construction projects in karst environments. To enhance structural stability, pile foundations are commonly used in karst areas, as they effectively transfer the superstructure loads to the bearing stratum at the pile tip as well as to the surrounding soil and rock layers [4]. However, the complex geological conditions in karst areas result in load transfer mechanisms and paths that differ markedly from those observed in non-karst regions.

Currently, most foundation structures in karst areas adopt single-pile foundations. Although the structural form of single-pile foundations is relatively simple, their bearing behavior is significantly influenced by the complex geological conditions. Based on numerical simulation results, Dai et al. [5] revealed the effects of karst cave number, spacing, and size on the additional deformation of pile foundations. Zhang et al. [6] primarily studied the impact of fully filled karst caves on pile foundation design. The results indicated that the elastic modulus of the karst cave infill soil had the greatest influence on single-pile bearing capacity, while the effects of Poisson’s ratio, friction angle, and density were relatively minor. Li et al. [7] analyzed the effects of pile diameter, the ratio of top plate thickness to pile diameter, and the ratio of cave width to pile diameter on the stability of foundations under karst conditions. The results indicated that the ratio of top plate thickness to pile diameter was a key factor governing pile foundation stability. Gotman et al. [8] developed a numerical method for calculating the bearing capacity of bridge pile foundations. The calculation results showed that pile bearing capacity, settlement, and stiffness coefficient can be determined based on foundation geometry, pile load, karst cave roof thickness, and cavity diameter. Sheng et al. [9] explored the bearing characteristics of pile foundations through physical model tests and finite element analysis. According to finite element analysis, an increase in pile head load leads to the formation of an arched tensile damage zone above the karst cave, which may ultimately induce rock stratum failure and collapse. Taking the Guizhou viaduct pile foundation project as a case, Zhao et al. [10] investigated the influence of karst caves on the bearing capacity characteristics of bridge pile foundations through numerical simulation. The numerical results indicated that the presence of karst caves reduces the bearing capacity of bridge pile foundations, with the degree of influence varying according to their relative position.

A pile group foundation [11] is an integrated foundation system consisting of multiple piles and a pile cap. As a complex system, the load transfer paths of a pile group foundation are influenced by both external conditions and internal factors, including the pile group effect [12], pile spacing [13], pile diameter [14], and pile length [15]. Based on full-scale lateral load tests of pile groups in hard clay, Rollins et al. [16] conducted inverse analyses, classified pile behavior categories, and established p-multiplier and normalised pile spacing curves. Based on negative friction model tests of pile group foundations, Huang et al. [17] studied the variations in pile stress, pile head displacement, and layered soil settlement under different additional load conditions. Based on the nonlinear Winkler spring beam model, Fayyazi et al. [18] established pile group numerical models to analyze the transverse mechanical response of pile group foundations and assess the effects of pile spacing and pile head conditions on the pile group effect coefficient. Ladhane et al. [19] developed a three-dimensional finite element program for dynamic analysis of pile groups and solved displacement responses with the implicit Newmark–beta method, thereby examining the effects of pile spacing, number of piles, pile arrangement, and soil modulus through parametric analysis. Fattah et al. [20] investigated the relative contributions of installation method, relative density, soil plug removal, pile length-to-diameter ratio, as well as shaft friction and end resistance to ultimate bearing capacity. Through FLAC3D numerical simulations, He et al. [21] constructed composite pile group foundation models under varying pile length conditions and analyzed the influence of pile–soil interaction on the pile group effect.

As is well known, the pile group effect on the bearing capacity of pile group foundations is primarily manifested in the influence of pile side friction and pile end resistance [22]. Norkus et al. [23] investigated the bearing capacity and deformation behavior of pile group foundations in dense sand under axial static loading, analyzed the effects of two-dimensional and three-dimensional pile spacing, and verified existing prediction models. The results indicated that pile foundation bearing capacity is dominated by end resistance, and that the pile group effect coefficient *η* increases as pile spacing decreases. Memar et al. [24] conducted experimental investigations on shaft friction in pile group foundations, comparing different pile cross-sections under varying pile arrangements, spacing, and sand relative density. The results indicated that shaft friction in pile group foundations varies with the pile cross-section. For pile group foundations, shaft friction resistance is typically calculated through the layered summation method [25]. Specifically, the ultimate shaft friction of a single pile in each soil layer is first determined and then multiplied by the pile group effect coefficient to obtain the total shaft friction resistance of the pile group. Similarly, the pile end resistance of a single pile is calculated and subsequently multiplied by the pile group effect coefficient to determine the total pile end resistance of the pile group. Commonly used approaches include the limit equilibrium method [26,27] and the elastic theory method [28]. With ongoing advances in theoretical frameworks and analytical techniques, pile foundations are increasingly applied in karst areas. However, complex geological and topographical conditions have resulted in a continued lack of comprehensive studies on the bearing behavior of pile foundations in karst environments.

As a result, considerable studies [29,30] have been focused on the bearing characteristics and the evolution of mechanical behavior of pile foundations in karst areas. Taking the Guangxi Nanyu High-Speed Railway project as a case study, He et al. [31] conducted an in-depth investigation of pile group bearing capacity through indoor model tests. The results showed that, as the number of karst caves increases, the bearing mechanism of pile foundations tends to shift from friction-controlled to end-bearing behavior. Li et al. [32] investigated bearing capacity and settlement behavior when beaded karst caves are located in different regions of pile group foundations. The results showed that pile foundations exhibit higher ultimate bearing capacity and smaller settlement when karst caves are located beneath the centre of the pile group. In summary, existing studies have primarily examined the influence of karst caves on pile group bearing capacity through numerical simulations or physical model tests, while assuming idealised cave positions. However, karst cave positions are far more complex in practical engineering conditions, including partial penetration of pile groups through caves, sidewall-adjacent caves, and caves offset beneath pile foundations. This paper, based on the pile foundation project at the Baiyun District Science and Technology Manufacturing Park in Guangzhou, explores the evolution of the ultimate bearing capacity and bearing characteristics of pile group foundations under offset karst cave conditions. Based on karst cave failure theory, the upper limit theorem of limit analysis, and pile group effect theory, a method for calculating the ultimate bearing capacity of pile group foundations under offset karst cave conditions is systematically derived and validated through field monitoring and numerical simulations.

## 2 Calculation of the ultimate bearing capacity of pile group foundations under underlying offset cavity conditions

The intricate interactions between piles and the surrounding soil are created by the complex geological conditions of karst terrain, leading to the formation of several foundation types, such as end bearing piles [33], friction piles [34], end bearing friction piles [35], and friction end bearing piles [36]. When the underlying bedrock is characterized with high bearing capacity, pile foundations in karst regions are typically designed as friction end bearing piles. The total bearing capacity of a pile foundation is determined with both side friction and end resistance. Side friction is generated by the interaction between the pile surface and adjacent soil, while end resistance is governed with the strength of the bedrock beneath the pile tip. Since pile group foundations are composed of multiple interacting piles, their bearing performance is further influenced with the pile group effect. Therefore, accurate evaluation of the ultimate bearing capacity of pile group foundations requires that side friction, end resistance, and the influence of pile group interaction all be taken into consideration.

**Fig 1:**
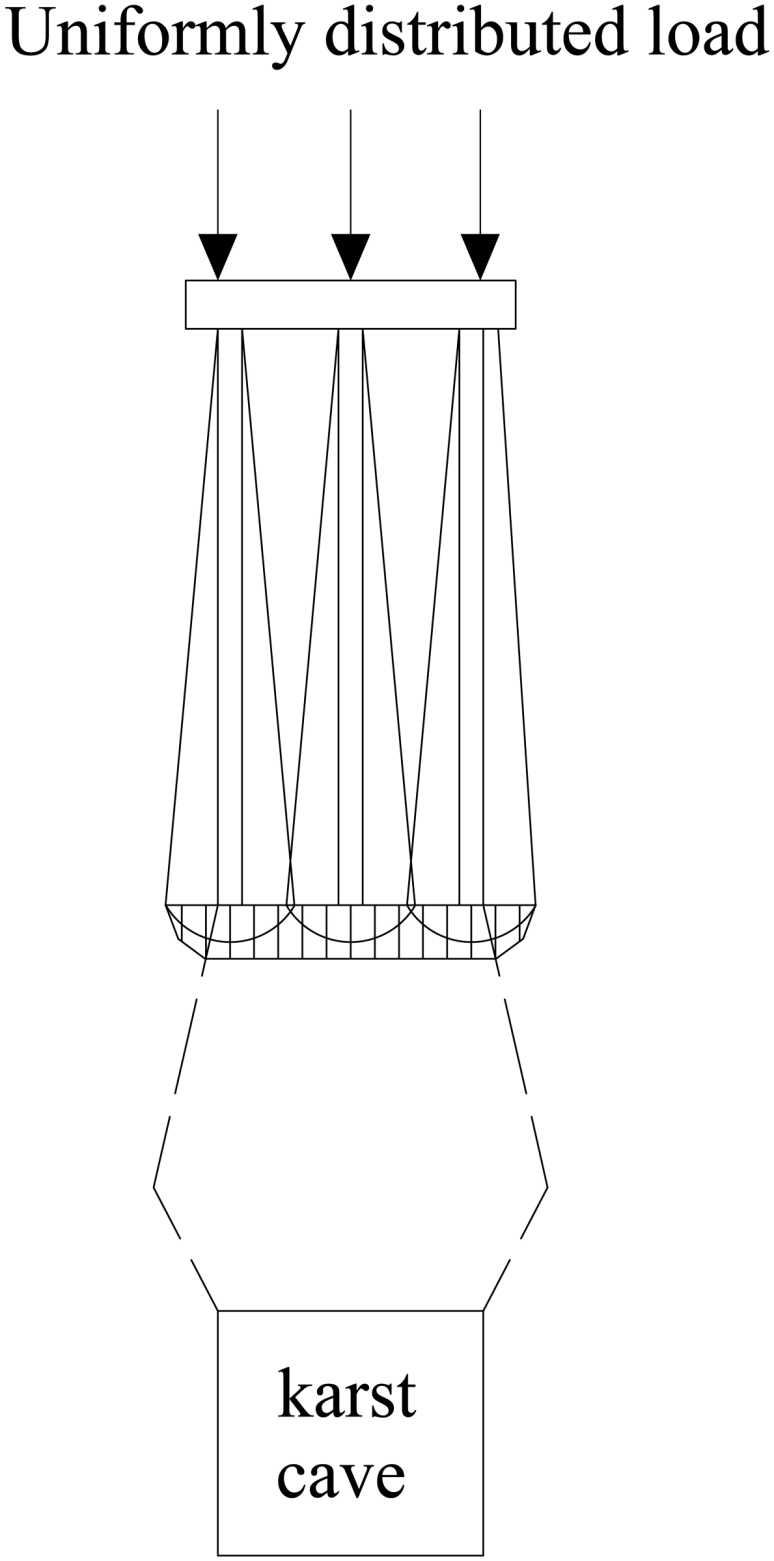
Punching shear failure mode.

**Fig 2:**
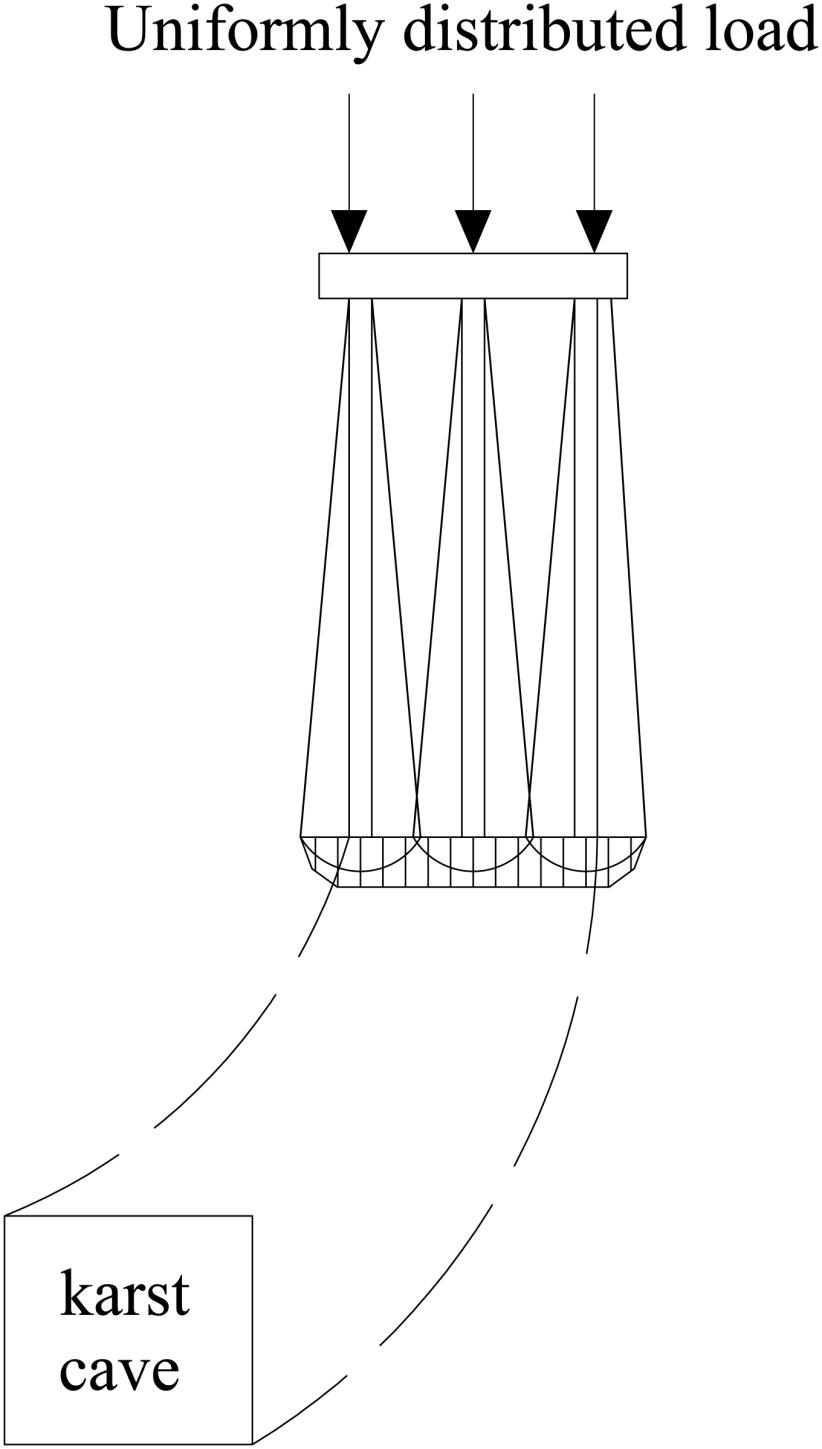
Double logarithmic spiral failure mode (The cave shifts to the right).

**Fig 3:**
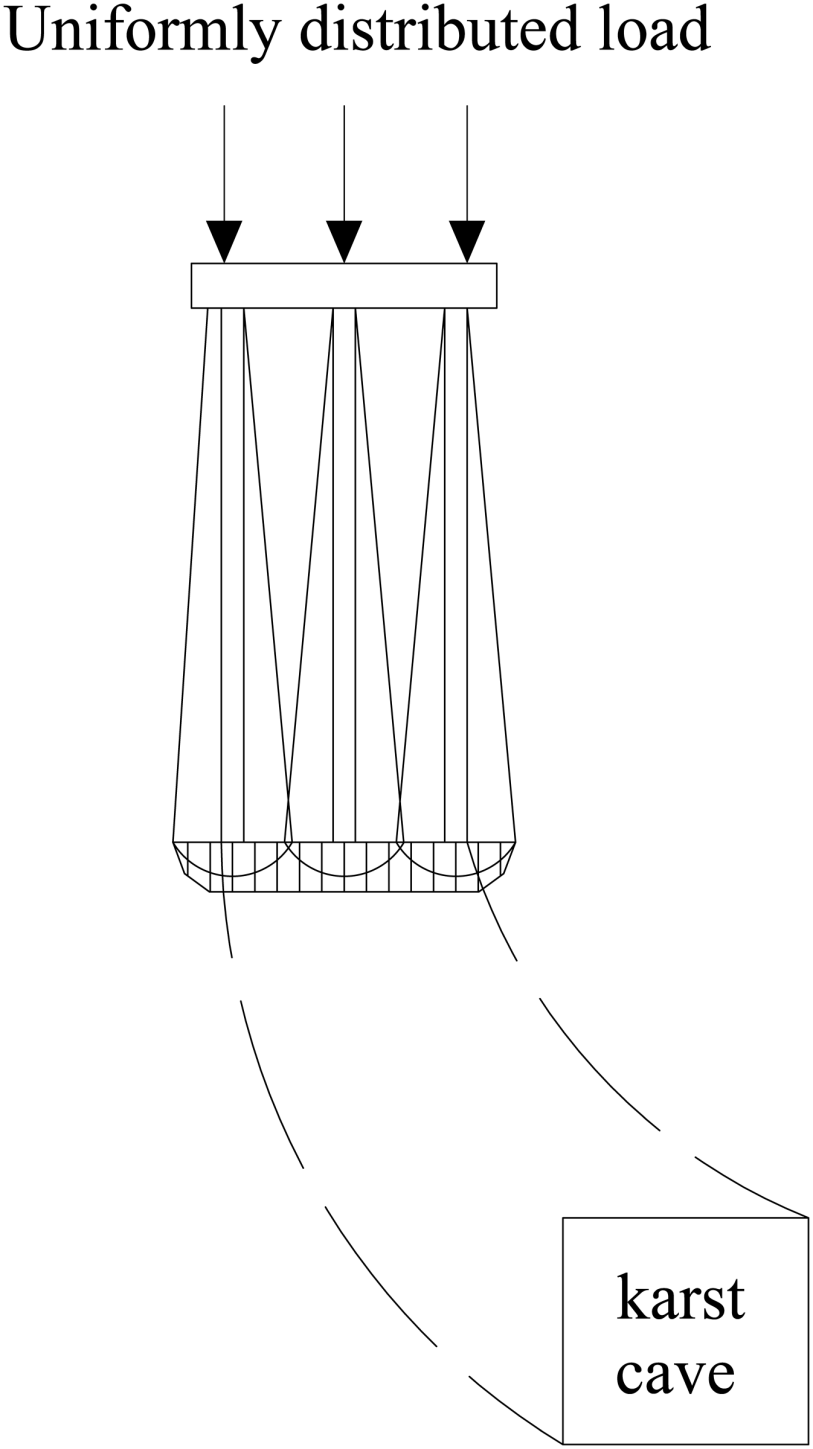
Double logarithmic spiral failure mode (The cave shifts to the left).

**Fig 4:**
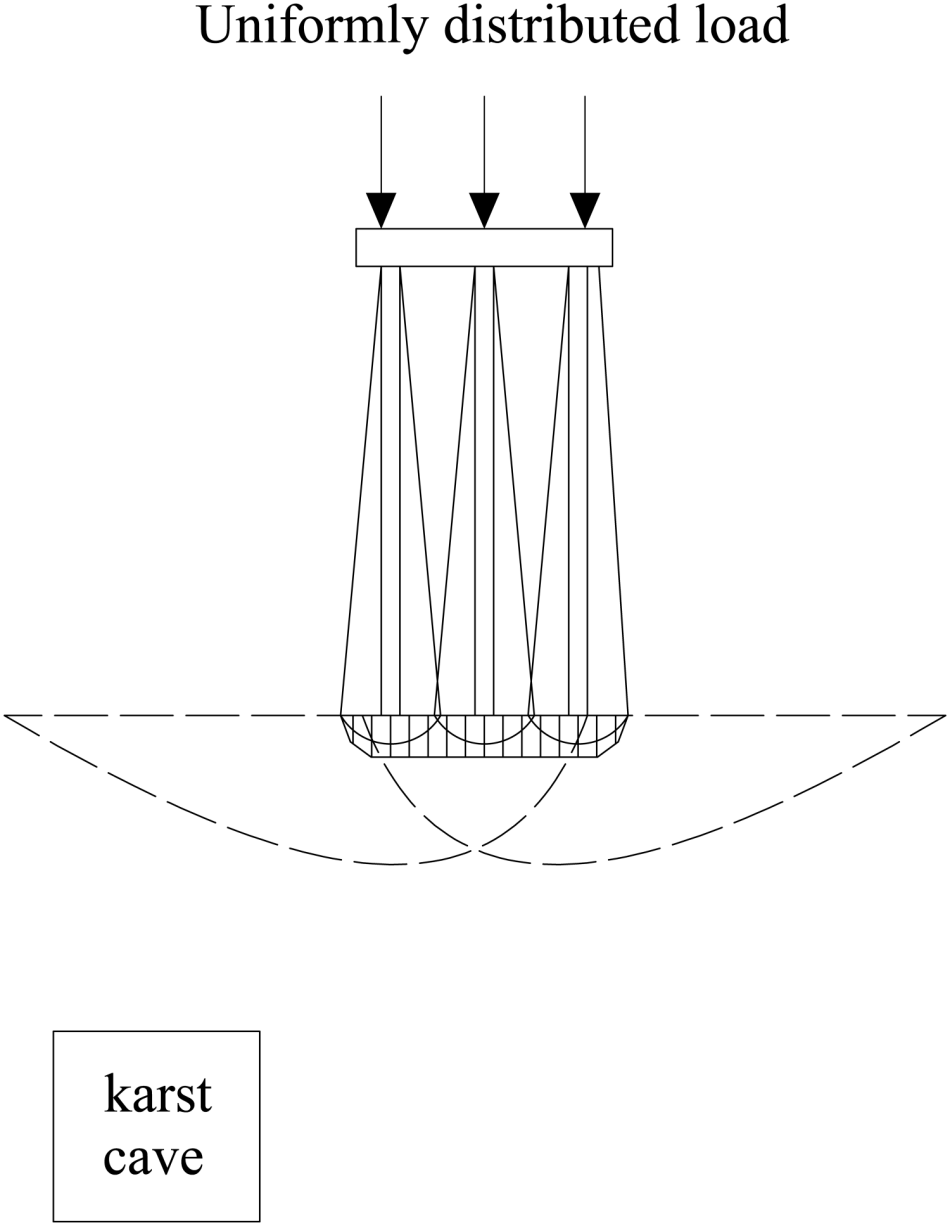
Prandtl destruction mode.

The relative position of the underlying karst cavity and the pile group leads to differences in stress distribution within the pile group foundation and in the bedrock failure mode. Based on single-pile and pile-group model tests, Lei et al. [37] identified the bearing mechanism of the rock layer at the pile tip under underlying karst cavity conditions and established a corresponding method for calculating the ultimate bearing capacity. The results show that when punching failure occurs in the rock layer at the pile tip, the resulting failure mass is equivalent to that of a large single pile. As shown in Figs 1–4, punching failure occurs in the bedrock when the karst cavity is located directly beneath the pile group. As the karst cavity position shifts, the bedrock failure mode transitions from punching failure [38] to a double-logarithmic spiral failure mode [39]. However, when the offset distance of the underlying karst cavity exceeds the threshold of its influence range, the instability failure of the pile foundation follows the Prandtl failure mode [40].

### 2.1 Solution of pile group effect

The interaction among piles and surrounding soil that alters load transfer within a group is described as the pile group effect. Side friction is reduced and resistance is concentrated at the pile tips with overlapping stress fields. Foundation capacity is strongly influenced by this effect. In practice, the bearing capacity of pile group is taken as the product of the capacity of a single pile, the number of piles in the group, and a pile group effect coefficient [41]. The equation is given as follows.


$$W_{U}=a⬝P_{U}⬝\eta$$ (1)

where *W*_*U*_ represents the ultimate bearing capacity of pile groups, *P*_*U*_ is the ultimate bearing capacity of single piles, *a* is the number of piles in the pile group foundation, and *η* is the pile group effect coefficient.

The pile group effect is currently evaluated by several approaches, including the entity perimeter method [42], the Converse Labarre method [43], the Seiler Keeney method [44], the partial coefficient method [45], and the stress superposition method [46]. The stress superposition method takes into account the effects of pile spacing, number of piles, pile length, and the properties of the surrounding soil and rock on the mechanical behavior of pile groups under karst conditions. To this end, the pile group coefficient is defined as the group bearing capacity divided by the product of the single pile capacity and the number of piles.


$$\eta =\frac{\text{1}}{1+\lambda }$$ (2)

$$\lambda =2A_{\text{1}}\frac{m-1}{m}+2A_{\text{2}}\frac{n-1}{n}+4A_{\text{3}}\frac{\left(m-1\right)\left(n-1\right)}{mn}$$ (3)

$$A_{\text{1}}=\frac{\text{1}}{3r_{\text{1}}}-\frac{\text{1}}{2ltan\varphi }$$ (4)

$$A_{\text{2}}=\frac{\text{1}}{3r_{\text{2}}}-\frac{\text{1}}{2ltan\varphi }$$ (5)

$$A_{\text{3}}=\frac{\text{1}}{3\sqrt{r_{\text{1}}^{\text{2}}+r_{\text{2}}^{\text{2}}}}-\frac{\text{1}}{2ltan\varphi }$$ (6)

where *λ* denotes the average reduction factor of the pile group foundation, *m* is the number of piles arranged transversely within the group, and *n* is the number of piles arranged longitudinally. *A*₁ represents the reduction factor caused by overlapping stress between adjacent piles in the transverse direction, *A*₂ represents the reduction factor due to overlapping stress between adjacent piles in the longitudinal direction, and *A*₃ represents the reduction factor arising from diagonal stress overlap among piles. *φ* is the weighted average internal friction angle of all soil layers along the pile shaft. *l* denotes the pile length, *r*₁ is the transverse pile spacing, and *r*₂ is the longitudinal pile spacing.

### 2.2 Solution of pile tip resistance

**Fig 5:**
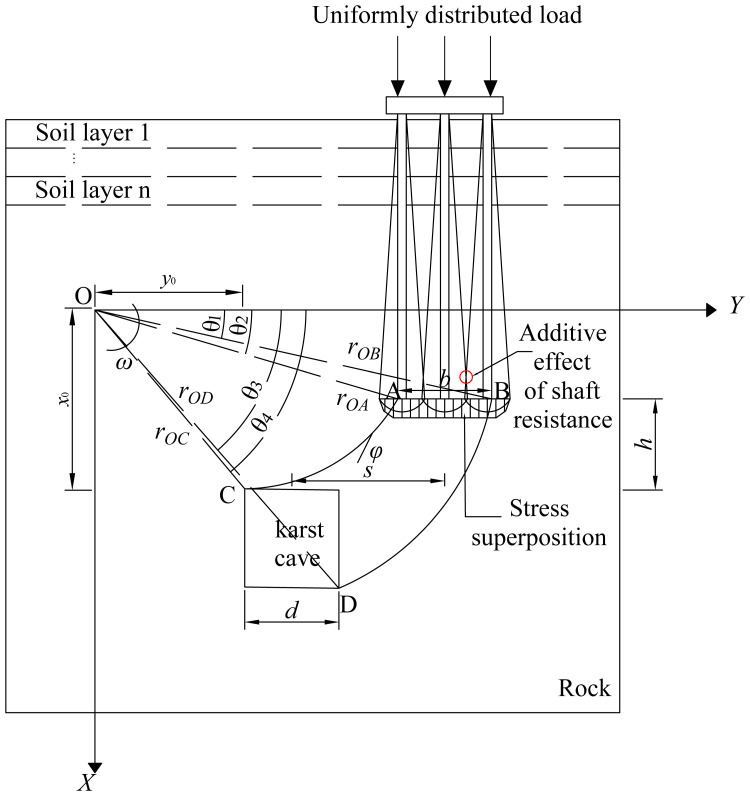
Schematic diagram of double logarithmic spiral failure mode.

The determination of pile tip resistance in a pile group is complex because group interaction causes the tip loads of individual piles to produce overlapping stress fields that concentrate stresses in the bearing stratum. Fig 5 illustrates that this concentration promotes a logarithmic spiral failure of the bedrock. In the schematic, AC and BD are logarithmic spiral curves, *b* denotes the extent of stress concentration beneath the pile tips, *d* is the cavity width, *h* is the bedrock thickness, *s* is the offset of the cavity, O is the center of the spiral, the coordinates of point C are given by (*x₀, y₀*), and *θ*₁*, θ*₂*, θ*₃, and *θ*₄ are the angles between OB*,* OA*,* OD, and OC and the y axis, respectively.

To determine the critical bedrock failure load, the following basic assumptions are adopted. Rock mass failure is assumed to follow the Mohr–Coulomb yield criterion, the material is isotropic, and the associated flow rule is satisfied. Parameter *b* is defined as the width of the equivalent stress concentration zone at the pile end plane. Parameter *d* represents the span of the karst cavity. Previous studies [47–49] have shown that the stress-affected zone at the bottom can be approximated by the geometry of the foundation when calculating the ultimate bearing capacity of a strip foundation. Based on foundation load distribution results, Fattah et al. [50] reported that shifts in karst cavity position produce consistent settlement and bearing capacity trends in both pile and strip foundations. Therefore, in the theoretical derivation of the ultimate bearing capacity of a pile foundation under karst cavity offset conditions, the stress concentration zone at the pile base is assumed to be consistent with that of a strip foundation. The width of the stress concentration zone at the pile base is taken as *b*. To facilitate theoretical and analytical solutions, studies [26] typically assume that the equivalent stress concentration range at the base of a strip foundation equals the karst cavity span, that is, *b* = *d*. Because strip and pile group foundations exhibit similar settlement and bearing behaviour, the assumption *b* = *d* is also adopted to simplify the analysis. As shown in Fig 2, the relevant geometric parameters can be obtained by the following equations.


$$r_{OA}=\frac{bsin\theta_{\text{1}}}{sin\left(\theta_{\text{2}}-\theta_{\text{1}}\right)}$$ (7)

$$r_{OB}=\frac{bsin\theta_{\text{2}}}{sin\left(\theta_{\text{2}}-\theta_{\text{1}}\right)}$$ (8)

$$x_{\text{0}}=r_{OB}sin\theta_{\text{2}}+h\text{\textbackslash{}hspace\{0.17em}\;\}y_{\text{0}}=r_{OA}cos\theta_{\text{2}}-eF_{AC}=\frac{bsin\theta_{\text{1}}}{sin\left(\theta_{\text{2}}-\theta_{\text{1}}\right)}⬝e^{\left[\left(\theta -\theta_{\text{2}}\right)tan\varphi \right]}$$ (9)

$$y_{\text{0}}=r_{OA}cos\theta_{\text{2}}-s$$ (10)

$$r_{OC}=\sqrt{x_{\text{0}}^{\text{2}}+y_{\text{0}}^{\text{2}}}$$ (11)

$$r_{OD}=\sqrt{(x_{\text{0}}+d{)}^{\text{2}}+(y_{\text{0}}+d{)}^{\text{2}}}$$ (12)

The logarithmic spiral failure surface is rotated about point O at a constant angular velocity *ω*, and the associated flow rule is satisfied by this motion. At any point on the slip surface, the angle between the velocity direction and the slip surface tangent is defined as being equal to the internal friction angle *φ*. Accordingly, the slip lines *F*_*AC*_ and *F*_*BD*_ are logarithmic spirals in polar coordinates centred at O, with *F*_*AC*_ expressed as follows.


$$F_{AC}=\frac{bsin\theta_{\text{1}}}{sin\left(\theta_{\text{2}}-\theta_{\text{1}}\right)}⬝e^{\left[\left(\theta -\theta_{\text{2}}\right)tan\varphi \right]}$$ (13)

Similarly, *F*_*BD*_, *r*_*OC*_, and *r*_*OD*_ can be obtained using the following equations.


$$F_{BD}=\frac{bsin\theta_{\text{2}}}{sin\left(\theta_{\text{2}}-\theta_{\text{1}}\right)}⬝e^{\left[\left(\theta -\theta_{\text{1}}\right)tan\varphi \right]}$$ (14)

$$r_{OC}=r_{OA}⬝e^{\left[\left(\theta_{\text{4}}-\theta_{\text{2}}\right)tan\varphi \right]}$$ (15)

$$r_{OD}=r_{OB}⬝e^{\left[\left(\theta_{\text{3}}-\theta_{\text{1}}\right)tan\varphi \right]}$$ (16)

Therefore, the work done by the external force can be expressed as follows.


$$W=\frac{\text{1}}{\text{2}}Q_{P}⬝\omega ⬝{\left(\frac{bsin\theta_{\text{2}}}{sin(\theta_{\text{2}}-\theta_{\text{1}})}\right)}^{\text{2}}⬝\left(1-\frac{sin^{\text{2}}\theta_{\text{1}}}{sin^{\text{2}}\theta_{\text{2}}}\right)$$ (17)

where *Q*_*P*_ is the ultimate bearing capacity of bedrock.

The internal energy dissipation is given as follows.


$$D_{AC}=\int_{\theta_{\text{2}}}^{\theta_{\text{4}}}c\frac{F_{AC}d\theta }{cos\varphi }\omega ⬝F_{AC}⬝cos\varphi =\omega ⬝c⬝{\left(\frac{bsin\theta_{\text{1}}}{sin(\theta_{\text{2}}-\theta_{\text{1}})}\right)}^{\text{2}}⬝\frac{e^{\left[2\left(\theta_{\text{4}}-\theta_{\text{2}}\right)tan\varphi \right]}-1}{2tan\varphi }$$ (18)

$$D_{BD}=\int_{\theta_{\text{1}}}^{\theta_{\text{3}}}c\frac{F_{BD}d\theta }{cos\varphi }\omega ⬝F_{BD}⬝cos\varphi =\omega ⬝c⬝{\left(\frac{bsin\theta_{\text{2}}}{sin(\theta_{\text{2}}-\theta_{\text{1}})}\right)}^{\text{2}}⬝\frac{e^{\left[2\left(\theta_{\text{3}}-\theta_{\text{1}}\right)tan\varphi \right]}-1}{2tan\varphi }$$ (19)

where *c* is the rock cohesion.

According to the upper limit principle of limit analysis, we can get the equation.


$$W=D_{AC}+D_{BD}$$ (20)

Substituting Equations (17), (18), and (19) into (20), we can get the equation.


$$\begin{array}{c} \frac{\text{1}}{\text{2}}Q_{P}\omega {\left(\frac{bsin\theta_{\text{2}}}{sin\left(\theta_{\text{2}}-\theta_{\text{1}}\right)}\right)}^{\text{2}}\left(1-\frac{{sin}^{\text{2}}\theta_{\text{1}}}{{sin}^{\text{2}}\theta_{\text{2}}}\right)=\omega c{\left(\frac{bsin\theta_{\text{1}}}{sin\left(\theta_{\text{2}}-\theta_{\text{1}}\right)}\right)}^{\text{2}}\frac{e^{2(\theta_{\text{4}}-\theta_{\text{2}})tan\varphi }-1}{2tan\varphi } \end{array}$$ (21)

Eliminating the common factor *ω* from both sides and simplifying, we can get the equation.


$$Q_{P}=\frac{\left({\left(\frac{bsin\theta_{\text{1}}}{sin\left(\theta_{\text{2}}-\theta_{\text{1}}\right)}\right)}^{\text{2}}⬝\frac{e^{\left[2\left(\theta_{\text{4}}-\theta_{\text{2}}\right)tan\varphi \right]}-1}{2tan\varphi }+{\left(\frac{bsin\;\theta_{\text{2}}}{sin\;\left(\theta_{\text{2}}-\theta_{\text{1}}\right)}\right)}^{\text{2}}⬝\frac{e^{\left[2\left(\theta_{\text{3}}-\theta_{\text{1}}\right)tan\;\varphi \right]}-1}{2tan\;\varphi }\right)⬝c}{{\left(\frac{bsin\;\theta_{\text{2}}}{sin\;\left(\theta_{\text{2}}-\theta_{\text{1}}\right)}\right)}^{\text{2}}⬝\left(1-\frac{sin^{\text{2}}\theta_{\text{1}}}{sin^{\text{2}}\theta_{\text{2}}}\right)}$$ (22)

Simplifying Equation (22), the pile end resistance *Q*_*P*_ can be obtained as follows.


$$\begin{array}{c} Q_{P}=\frac{\left[{sin}^{\text{2}}\theta_{\text{1}}⬝\left(e^{\left[2\left(\theta_{\text{4}}-\theta_{\text{2}}\right)tan\varphi \right]}-1\right)+{sin}^{\text{2}}\theta_{\text{2}}⬝\left(e^{\left[2\left(\theta_{\text{3}}-\theta_{\text{1}}\right)tan\varphi \right]}-1\right)\right]⬝c}{2tan\varphi \left({sin}^{\text{2}}\theta_{\text{2}}-{sin}^{\text{2}}\theta_{\text{1}}\right)} \end{array}$$ (23)

Within the analytical framework of double logarithmic spiral failure for pile tip disturbance caused by karst, the potential failure surface is typically treated as a spiral curve that extends outward from the center of rotation. Its spatial morphology must satisfy both geometric and mechanical rationality. To preserve the fundamental characteristic of a failure surface that opens progressively from depth to the top, the maximum diameter of the failure curve in the upper region should exceed that in the deeper region. This condition allows the failure path to expand outward in a stable manner as the angle increases. The horizontal position of the center of rotation must be located within the bearing soil behind the pile foundation. It should not be situated in the ineffective zone in front of the pile or outside the pile body. This requirement ensures that the potential failure process develops as a rotational slip mode around the center and does not evolve into overturning, linear shear, or other failure types that are inconsistent with actual working conditions. The polar angle of the failure surface along the failure path should increase monotonically from depth to the top and remain within the first quadrant. This requirement allows the failure curve to achieve a reasonable spatial extension within the soil and to maintain continuity as it penetrates the pile tip region. It also prevents the formation of unreasonable morphologies such as reverse rotation. The geometric and mechanical constraints described above are presented in Equation (24).


$$\left\{ \begin{array}{c} @l@r_{OA}<r_{OB}\\ x_{o}-h>0\\ 0<\theta_{\text{1}}<\theta_{\text{1}}<\frac{\pi }{\text{2}} \end{array}\right.$$ (24)

Under constraint Equation (24), the ultimate resistance at the pile tip of the pile group foundation is computed, and the optimal solution is obtained with MATLAB.

### 2.3 Pile side friction

The side friction of a pile group foundation is determined by summing the side friction of each pile in the group while accounting for the pile group effect coefficient. For an individual pile within the group, the side friction is calculated using the following expression.


$$Q_{S}=\int_{\text{0}}^{l}\tau_{i}\left(z\right)S\left(z\right)dz$$ (25)

where *Q*_*S*_ represents the total side friction of a single pile, *L* is the pile length, *τ*_*i*_*(z)* denotes the side friction at depth *z* in the i-th soil layer, and *S(z)* is the surface area of the pile in contact with the surrounding soil at that depth.


$$\tau_{i}\left(z\right)=\gamma_{i}zk_{i}$$ (26)

where *γ*_*i*_ denotes the unit weight of the corresponding rock or soil layer, *z* is the depth of the pile within the ground, and *k*_*i*_ is the friction coefficient of that layer.

Therefore, the total side friction resistance of a pile group foundation is obtained by multiplying the side friction resistance of a single pile with the number of piles in the group and by the pile group effect coefficient, expressed as follows.


$$Q_{total}=a⬝Q_{S}⬝\eta$$ (27)

### 2.4 Solution of the ultimate bearing capacity of pile group foundation

The ultimate bearing capacity of a single pile is composed of two components: the end resistance and the side friction resistance, expressed as follows.


$$P_{U}=Q_{S}+Q_{P}$$ (28)

where *Q*_*S*_ denotes the side friction resistance, and *Q*_*P*_ represents the tip resistance of the pile.

The ultimate bearing capacity of a pile group foundation includes both side resistance and end resistance. With combining Equations 23 and 27, the ultimate bearing capacity of the pile group foundation can be expressed as follows.


$$\begin{array}{c} \begin{array}{c} W_{U}=\frac{\left[{sin}^{\text{2}}\theta_{\text{1}}⬝\left(e^{\left[2\left(\theta_{\text{4}}-\theta_{\text{2}}\right)tan\varphi \right]}-1\right)+{sin}^{\text{2}}\theta_{\text{2}}⬝\left(e^{\left[2\left(\theta_{\text{3}}-\theta_{\text{1}}\right)tan\varphi \right]}-1\right)\right]⬝c}{2tan\varphi \left({sin}^{\text{2}}\theta_{\text{2}}-{sin}^{\text{2}}\theta_{\text{1}}\right)}\\ \;\;\;+\;n⬝\eta ⬝\int_{\text{0}}^{l}\tau_{i}\left(z\right)S\left(z\right)dz \end{array} \end{array}$$ (29)

The double logarithmic spiral failure analysis framework proposed in this study represents a transitional failure model for describing the continuous evolution of failure modes in hollow rock foundations under load–displacement conditions. When the load–displacement distance is small, the failure path tends to concentrate directly beneath the pile tip, and the double logarithmic spiral curve degenerates into a punching shear–dominated failure mode. As the load–displacement distance increases, the failure path expands outwards towards the weaker side and develops into a typical double logarithmic spiral failure pattern. When the displacement distance increases further and reaches a critical level, the optimisation solution approaches the Prandtl failure mode under intact bedrock conditions.

## 3 Project overview and on-site monitoring

### 3.1 Project overview

The pile foundation project of the Science and Technology Manufacturing Park in Baiyun District, Guangzhou is used as the field case in this study. A typical karst setting with covered cavities is confirmed by site investigation. From top to bottom, the stratigraphy is composed of miscellaneous fill, silty soil, silty clay, and limestone. The miscellaneous fill has a thickness of 4–7 m, the silty soil 5–9 m, the silty clay 5–10 m, and the limestone 30–70 m. Cavities are found to occur at depths of 30–60 m. The physical param of the rock and soil layers are listed in Table 1.

**Table 1:** Geophysical param.

	*ρ*/kg·m^-3^	*E*/MPa	*c*/MPa	*v*	*φ*/°
Miscellaneous fill soil	1630	3.1	0.012	0.3	9
Silty soil	1760	4	0.017	0.31	13
Silty clay	1920	10.5	0.1	0.33	21
Limestone	2290	1500	6	0.25	34

**Fig 6:**
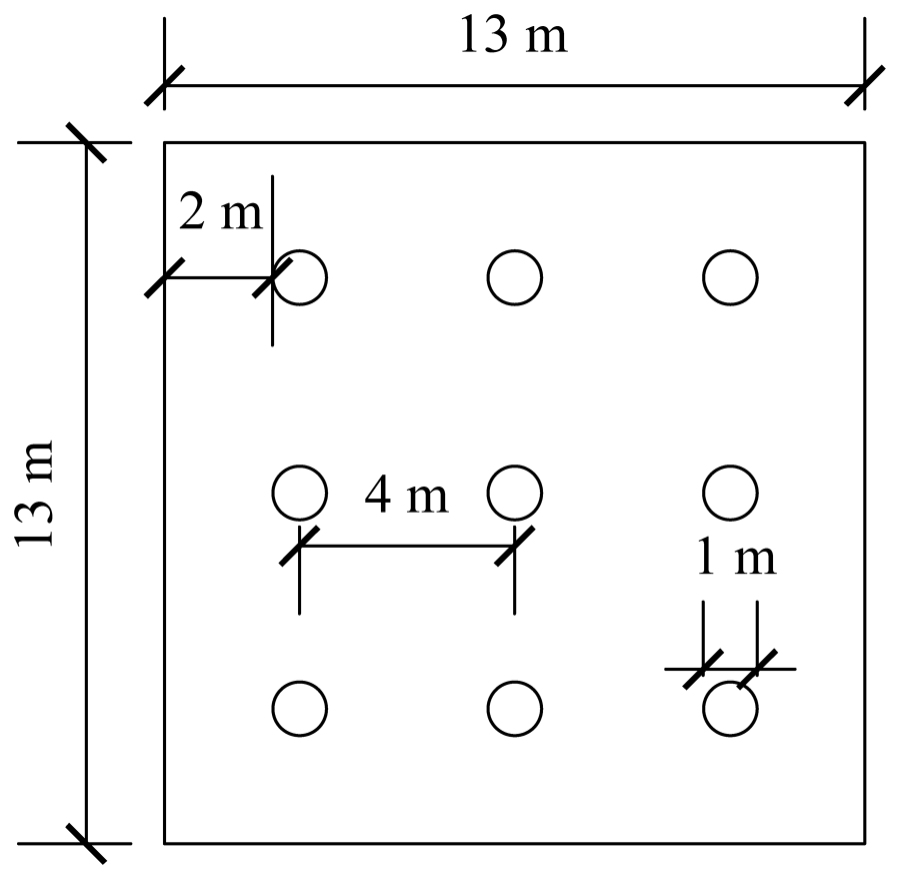
Schematic diagram of pile group foundation layout.

Underlying cavities were identified by geological exploration in 59 of 68 boreholes. According to the national standard ‘General Specification for Building and Municipal Foundations’ (GB 55003 2021), the borehole porosity is calculated as 86.76% and the karst rate along the borehole line is determined to be 21.33%, indicating that the site is characterized by strong karst development. The rock mass is classified as slightly weathered limestone with adequate strength, and sufficient bearing capacity can be provided by it. Therefore, a pile group with a 3 × 3 arrangement of friction end bearing piles is adopted. Across the site, pile lengths are designed to range from 18 to 40 m and pile diam are specified to range from 0.8 to 1.6 m. At the location selected for detailed study, the pile length is set at 21 m and the pile diameter is fixed at 1 m. The layout of the pile group foundation is shown in Fig 6.

### 3.2 Pile group foundation settlement monitoring

Load testing and monitoring were performed on five pile groups designated AZK03, AZK08, AZK10, AZK11, and AZK14 after construction was completed. For AZK03, a karst cavity is located about eight m below the pile toes, and its center is offset by about four m from the group center. For AZK08, the cavity is positioned about eight m below the pile toes with an offset of about eight m. AZK10 is characterized as a group in which the piles are designed to pass completely through a cavity, and the pile length is set at about thirty m. Beneath AZK11, cavities are distributed in a small and dispersed pattern. AZK14 is defined as a group in which only part of the piles is constructed to pass through a cavity.

**Fig 7:**
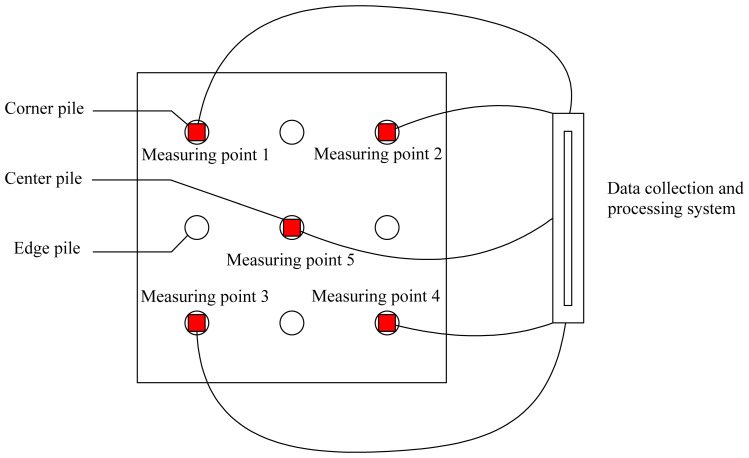
Monitoring diagram.

The settlement of the pile foundation is monitored through high-precision displacement sensors. As shown in Fig 4, the sensors are connected to a data acquisition and processing system to record real-time settlement data. The monitoring device is shown in Fig 7.

High-precision displacement sensors were installed at the four corner piles and the central pile of the pile group foundation for settlement monitoring. They were connected to a data acquisition and processing system. Differential settlement across the cap was tracked by the corner sensors, while the primary indicator of overall settlement was provided by the central sensor. Loading was applied by stacking high-density concrete blocks, which were lifted by crane and placed uniformly on the cap to simulate a uniform surface load. The design load was set at 700 kPa. Each loading increment was set equal to 20% of the design load. After each increment, the load was maintained for thirty minutes to allow settlement to be stabilized. Loading was stopped when the design load was reached or when the settlement rate was observed to show a sudden change or an excessive increase. Settlement magnitude and rate were recorded at every loading level. If no obvious change in the settlement growth rate was shown at the design load, loading was continued with smaller increments of 10%. Loading was stopped immediately when the settlement rate was observed to rise sharply, and the corresponding load was recorded after settlement stabilization was observed. The on-site monitoring data are shown in Tables 2–6.

**Table 2:** Monitoring results of AZK03 pile group foundation/mm.

Uniformly distributed Load/ kPa	Measuring point 1	Measuring point 2	Measuring point 3	Measuring point 4	Measuring point 5
140	8.15	7.63	7.95	8.09	7.67
280	16.67	15.61	16.26	16.55	15.69
420	24.54	22.98	23.94	24.37	23.10
560	32.55	30.49	31.76	32.33	30.65
700	40.27	37.72	39.29	40.00	37.91
770	44.71	41.88	43.62	44.41	42.09
840	49.73	46.58	48.52	49.39	46.82

**Table 3:** Monitoring results of AZK08 pile group foundation/mm.

Uniformly distributed load/ kPa	Measuring point 1	Measuring point 2	Measuring point 3	Measuring point 4	Measuring point 5
140	8.11	7.48	7.94	8.15	7.25
280	16.59	15.83	15.73	16.67	15.53
420	24.42	23.74	23.39	24.54	22.89
560	32.40	31.27	31.59	32.55	31.02
700	40.08	38.09	38.69	40.27	38.92
770	41.49	42.88	43.62	42.71	43.18
840	49.49	47.59	48.53	49.73	46.97
910	56.43	53.11	55.52	56.70	53.38

**Table 4:** Monitoring results of AZK10 pile group foundation/mm.

Uniformly distributed load/ kPa	Measuring point 1	Measuring point 2	Measuring point 3	Measuring point 4	Measuring point 5
140	10.67	10.63	10.73	10.68	10.62
280	18.92	18.85	19.05	18.94	18.81
420	26.61	26.69	26.82	26.56	26.63
560	34.42	34.19	34.56	34.35	34.21
700	42.95	43.12	43.38	43.25	42.99
770	47.23	47.15	47.65	47.41	47.20
840	52.44	52.34	52.76	52.55	52.23

**Table 5:** Monitoring results of AZK11 pile group foundation/mm.

Uniformly distributed load/ kPa	Measuring point 1	Measuring point 2	Measuring point 3	Measuring point 4	Measuring point 5
140	6.68	6.77	6.73	6.71	6.79
280	15.01	15.21	15.09	15.06	15.15
420	22.65	22.42	22.58	22.78	22.49
560	30.10	30.55	30.37	30.43	30.28
700	38.49	37.99	38.30	38.57	38.22
770	42.60	43.11	42.73	42.47	42.82
840	47.81	47.19	47.43	47.62	47.38
910	53.20	53.73	53.57	53.62	53.09

**Table 6:** Monitoring results of AZK14 pile group foundation/mm.

Uniformly distributed load/ kPa	Measuring point 1	Measuring point 2	Measuring point 3	Measuring point 4	Measuring point 5
140	8.81	8.29	8.64	8.81	8.35
280	16.50	15.53	16.18	16.50	15.61
420	24.98	23.51	24.49	24.98	23.63
560	33.11	31.16	32.46	33.11	31.32
700	41.10	38.68	40.29	41.10	38.88
770	45.37	42.70	44.48	45.37	42.92
840	50.51	47.54	49.52	50.51	47.79
910	56.85	53.51	55.74	56.85	53.79

The calculated results indicate that the ultimate bearing capacities of AZK03, AZK08, AZK10, AZK11, and AZK14 are 770 kPa, 840 kPa, 770 kPa, 840 kPa, and 840 kPa, respectively.

## 4 Model establishment and verification

### 4.1 Finite element model design

**Fig 8:**
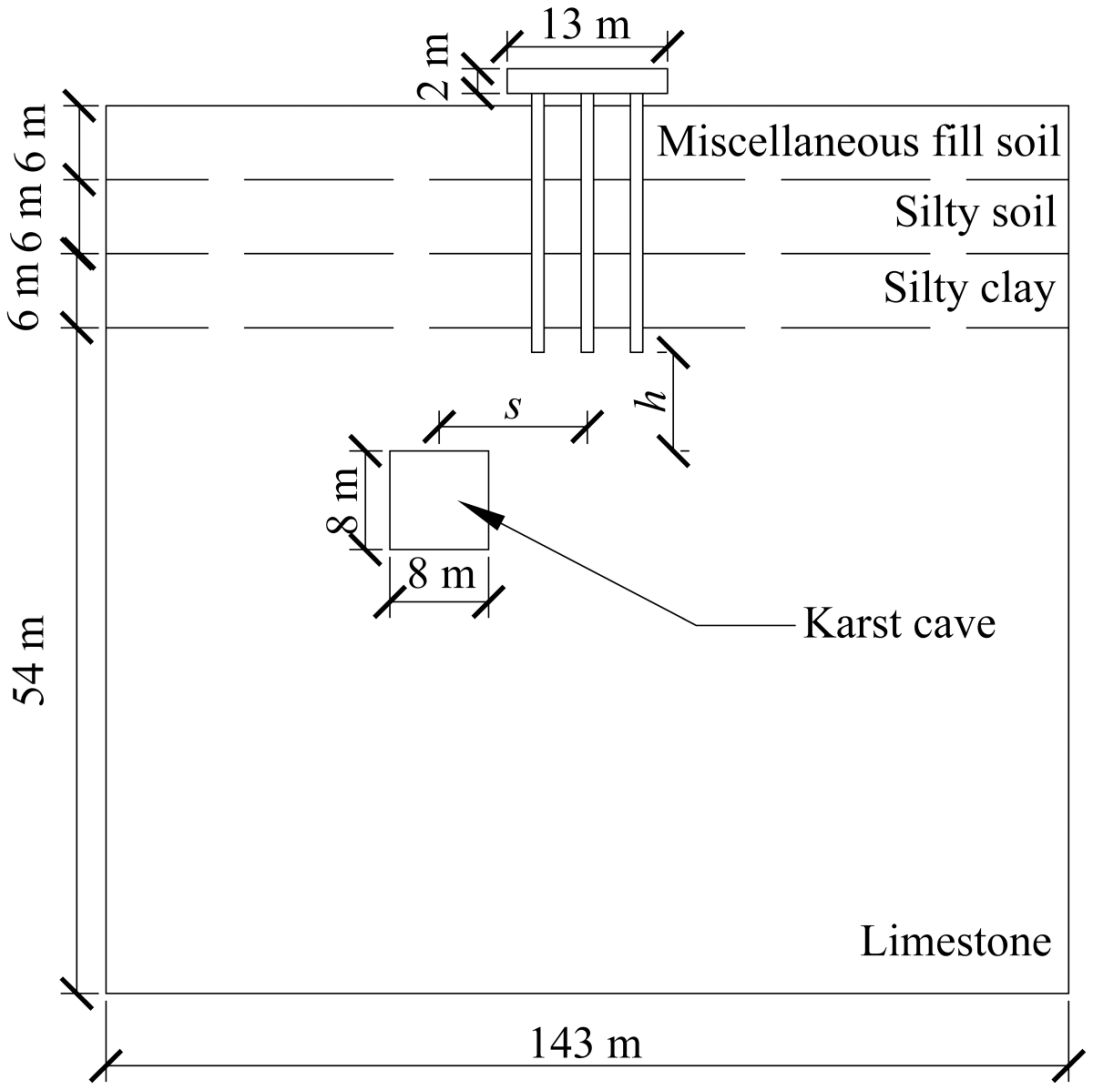
Model size diagram.

**Fig 9:**
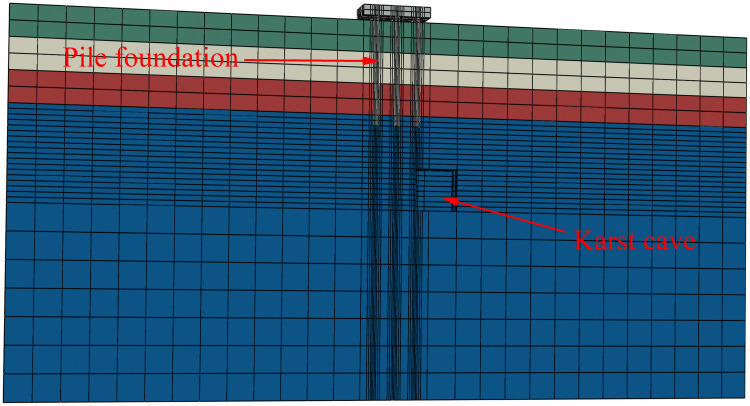
Model diagram.

A computational model for pile foundation engineering at sites AZK03 and AZK08 is developed using ABAQUS finite-element numerical simulation software. A model is a full-scale representation of the pile group foundation, with a pile cap size of 13 × 13 × 2 m, a pile length of 21 m, and a pile diameter of 1 m. The karst cavity location is consistent with field investigation results and lies 8–10 m below the pile base. For numerical stability and modelling efficiency, the karst cavity is assumed to be a regular cuboid with dimensions of 8 × 8 × 8 m. In addition, the soil width was set to five times the pile cap width, and the soil height to twice the combined height of the pile foundation and the karst cavity, resulting in a model size of 143 × 143 × 72 m. From top to bottom, the soil profile consists of 6 m of miscellaneous fill, 6 m of silty clay, a further 6 m of silty clay, and 54 m of limestone. The embedment depth of the pile group foundation into the rock is 2 m. Furthermore, local mesh refinement was applied to stress concentration regions, including the pile shaft, pile cap base, and karst cavity boundaries, to better capture stress and deformation gradients. Soil regions distant from the pile foundation and karst cavities were modelled using relatively coarse meshes to reduce computational cost. The model contains 59,148 soil elements and 1,880 pile group foundation elements, giving a total of 61,028 elements. The model configuration is shown in Figs 8 and 9.

**Fig 10:**
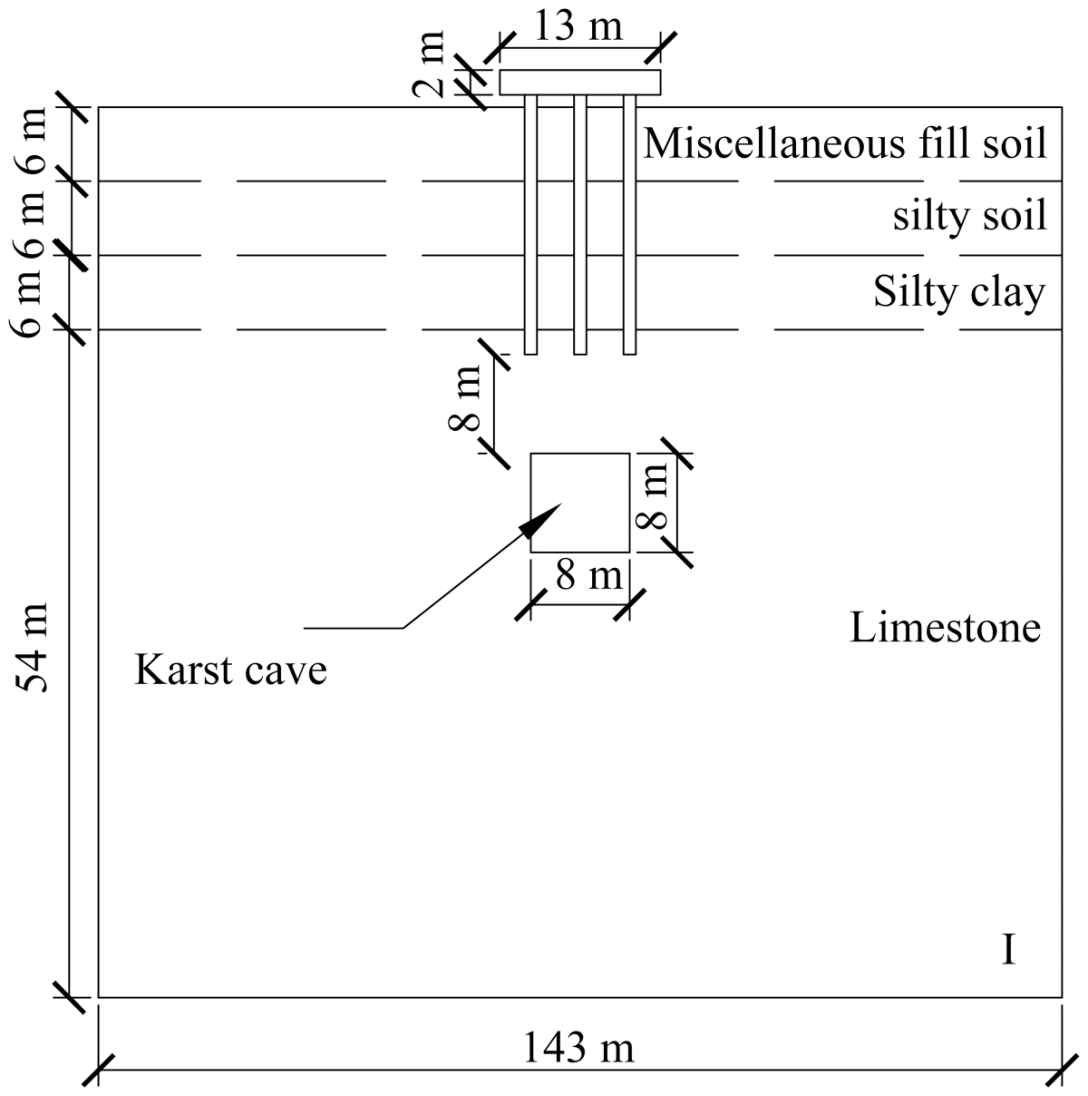
Working condition diagram of karst cave no-offset.

**Fig 11:**
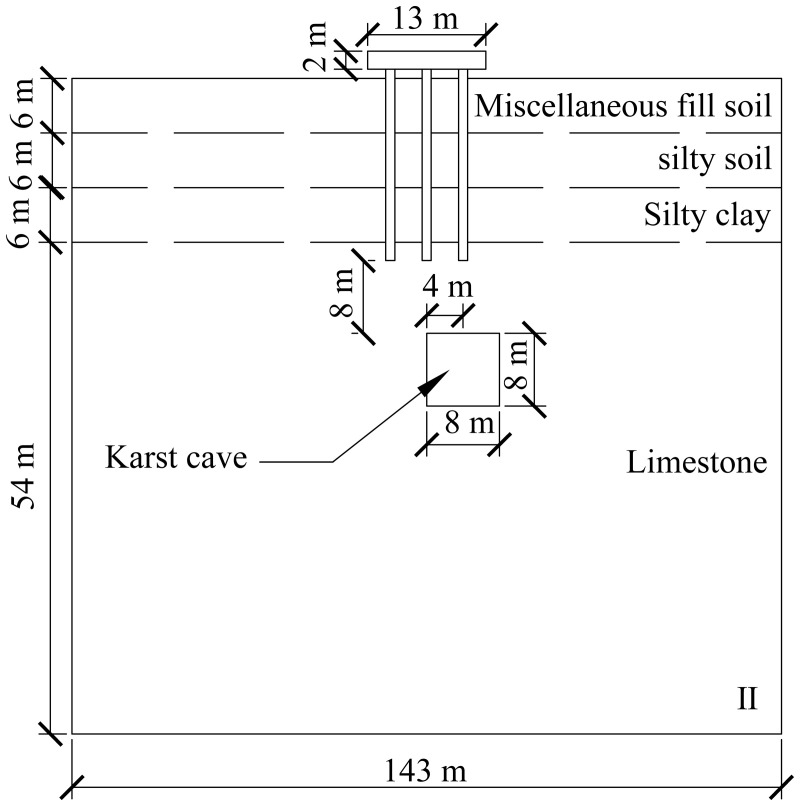
Working condition diagram of cave offset 4 m.

**Fig 12:**
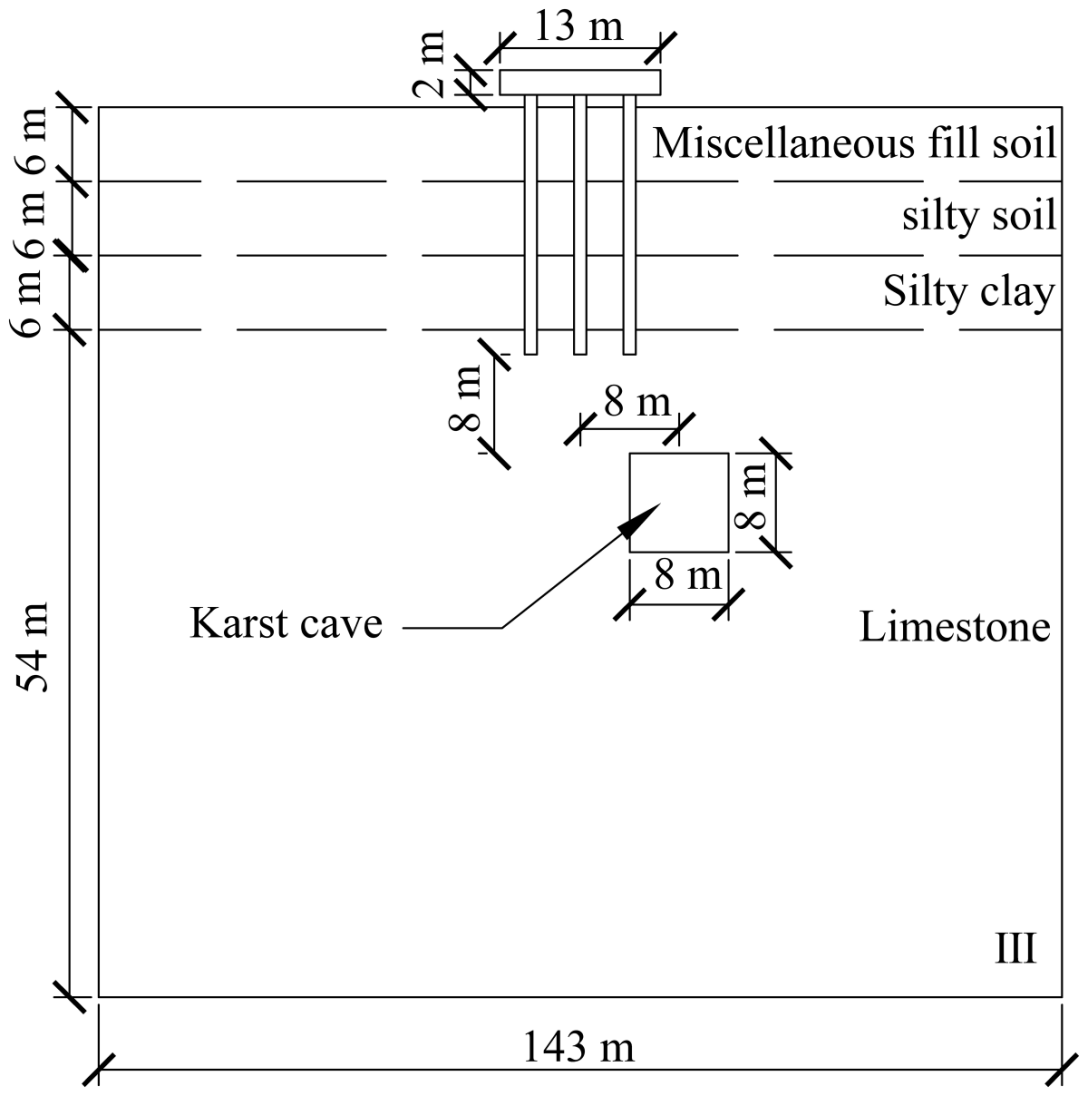
Working condition diagram of cave offset 8 m.

The specific numerical simulation conditions are shown in Figs 10–12. The thickness of the cavity roof and the bedrock at the pile tip is 8 m. The offset distance is defined as the lateral distance between the center of the cavity and the center of the pile group foundation. The scenario with no offset is designated as Case I, the scenario with a 4 m offset is designated as Case II, and the scenario with an 8 m offset is designated as Case III.

### 4.2 Material param and contact surface settings

Since elastic deformation, shear yield, and plastic flow are typically undergone by the rock and soil around the piles under external loading, a Mohr-Coulomb elastic-plastic constitutive model is adopted in the analysis. This model, which is grounded in Mohr-Coulomb strength theory, defines the shear yield criterion and the plastic flow rule through critical parameters such as the internal friction angle *φ* and cohesion *c*, enabling the capture of stress redistribution and the evolution of plastic deformation in the surrounding ground under load. The piles and the cap are modeled as a single linear elastic body, as their elastic limits are positioned far above the load levels used in the simulations. The specific physical and mechanical parameters are listed in Table 7.

**Table 7:** Relevant mechanical param of piles and surrounding rock and soil.

Name	*ρ*/kg·m^-3^	*ε*/MPa	*c*/MPa	*v*	*φ*/°
Miscellaneous fill soil	1630	3.1	0.012	0.3	9
Silty soil	1760	4	0.017	0.31	13
Silty clay	1920	10.5	0.1	0.33	21
Limestone	2290	1500	6	0.25	34
Pile (cap)	2400	30000	—	0.19	—

Given that the piles are much stronger than the surrounding rock and soil, the pile surface was defined as the primary contact and the rock and soil surface as the secondary contact. Tangential interaction was modeled with a penalty formulation using a friction coefficient of 0.35. Normal interaction was modeled as hard contact.

### 4.3 Boundary conditions and loading settings

Appropriate boundary conditions were imposed to reproduce the interaction between real soil and the pile group foundation. Normal constraints were applied to the side surfaces, which allowed vertical movement of the ground while limiting normal displacement. The bottom surface was fixed in all degrees of freedom, and the top surface was left free. A uniformly distributed load was employed because it is considered to represent the load-bearing behavior of pile groups in practice [51]. The model was loaded in increments of 200 kPa. During setup, the top surface of the cap was kinematically coupled to the geometric center of the cap so that the uniform load could be applied as an equivalent concentrated force.

### 4.4 Model validity verification

The accuracy of the numerical model was verified by comparing the simulation results with field monitoring data. Settlement of the pile group foundation under corresponding load conditions for both schemes served as the evaluation criterion. The comparison results are presented in Tables 8 and 9.

**Table 8:** AZK03 settlement data/mm.

Load/kPa	140	280	420	560	700	770	840
Numerical simulation	8.54	17.12	25.47	33.47	41.93	45.12	50.24
Actual monitoring	7.95	16.26	23.94	31.76	39.29	43.62	48.52
Relative error	7.42%	5.29%	6.39%	5.38%	6.72%	3.44%	3.54%

**Table 9:** AZK08 settlement data table/mm.

Load/kPa	140	280	420	560	700	840	910
Numerical simulation	8.48	16.42	24.17	32.94	40.54	51.95	58.13
Actual monitoring	7.94	15.73	23.39	31.59	38.69	48.53	55.52
Relative error	6.80%	4.39%	3.33%	4.27%	4.78%	7.05%	4.70%

As shown in Tables 8 and 9, larger settlements are predicted by the numerical simulation than are measured in the field. This discrepancy is attributed to the fact that each rock and soil layer is idealized as homogeneous in the model, while the actual ground is characterized by heterogeneity. Additionally, only pile-soil interaction is considered in the model, while complex pile interaction and local variability in soil properties are not captured. Although discrepancies are observed between the numerical simulation and the field measurements, the relative error is calculated to range from 3.33% to 7.42%, which is considered to be within a reasonable range.

### 4.5 Mesh convergence analysis

To ensure result accuracy and minimise the effects of mesh coarsening, a systematic mesh convergence analysis was performed. Mesh details are presented in Table 10.

**Table 10:** Mesh count and calculation results.

Mesh	21224	31112	61028	136664
Ultimate bearing capacity	880 kPa	890 kPa	900 kPa	905 kPa

**Fig 13:**
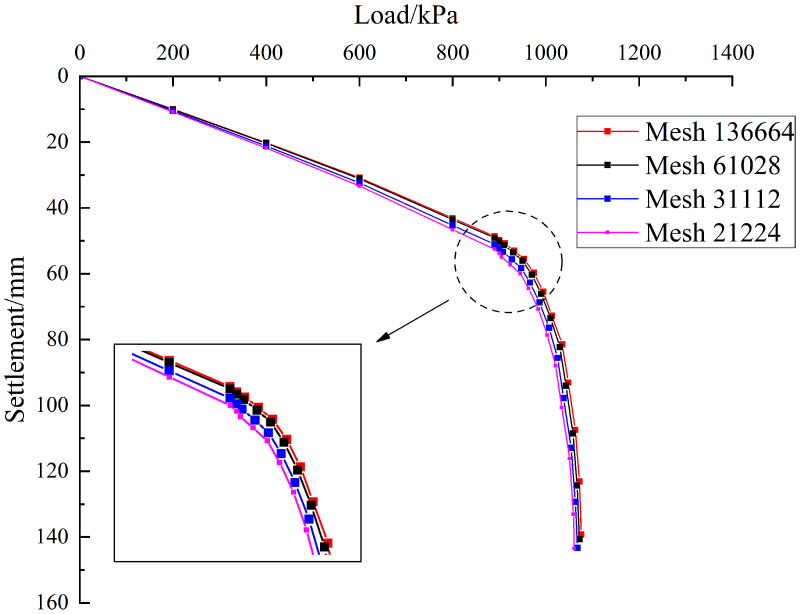
Load–settlement curves of pile group foundations with different mesh element numbers.

The feasibility of the current mesh discretisation will be verified by progressively refining the computational mesh and comparing simulation results across different mesh densities. As shown in Fig 13, the ultimate bearing capacity increases with the number of mesh elements, and the simulation results become closer to the measured values. Specifically, when the number of mesh elements is 21,224, 31,112, 61,028, and 136,664, the corresponding ultimate bearing capacities of the pile foundation are 880 kPa, 890 kPa, 900 kPa, and 905 kPa, respectively. It can be observed that, compared with the model containing 61,028 mesh elements, the model with 136,664 elements shows only a 0.5% increase, and the mechanical response curves exhibit nearly identical trends. In other words, the model with 61,028 mesh elements has effectively converged. Therefore, to reduce computational cost, the model with 61,028 mesh elements was adopted.

### 4.6 Analysis of numerical simulation results

**Fig 14:**
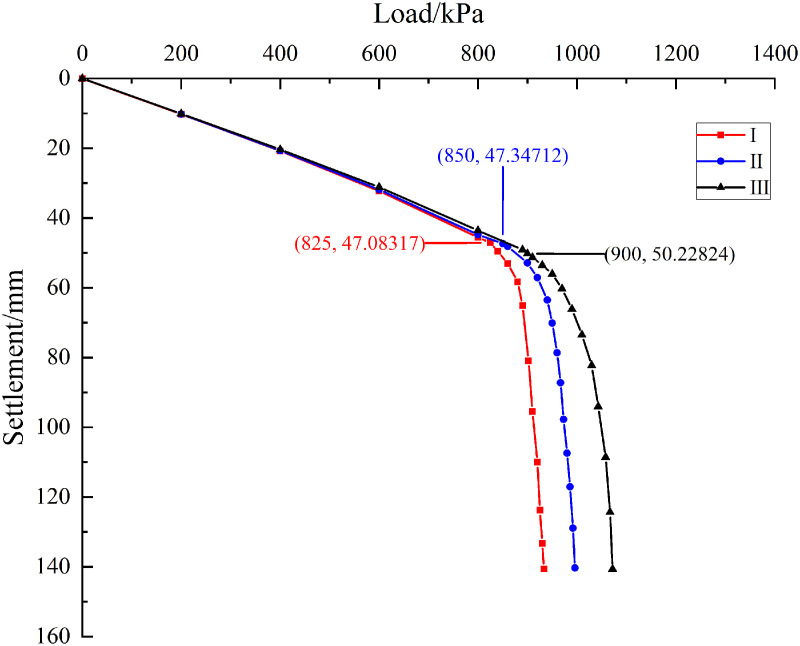
Load-settlement curve.

As shown in Fig 14, the load-settlement curves under the three conditions are initially observed to be linear, and are then transformed into nonlinear with rapid settlement once the ultimate bearing capacity of the pile group foundation is reached. In the linear stage, the three curves are found to be relatively similar, which indicates that the initial tangent stiffness of the system is maintained consistently. Different ultimate capacities are caused by different offsets of the underlying karst cavity, and therefore the inflection points where the curves enter the nonlinear stage are also varied. A distinct inflection point is observed at 825 kPa in Condition I, at 850 kPa in Condition II, and at 900 kPa in Condition III. According to the ‘Technical Specification for Testing of Building Foundation Pile’ (JGJ 106 2014), a pronounced steep drop in a load-settlement curve is interpreted to mean that the load at the start of this drop is taken as the ultimate load. On this basis, the ultimate bearing capacity is determined to be about 825 kPa in Condition I, about 850 kPa in Condition II, and about 900 kPa in Condition III. Relative to Condition I, the capacity in Condition III is increased by about 9.09%, and relative to Condition II, it is increased by about 5.88%. These results indicate that as the karst cavity offset is increased, the influence of the cavity is weakened and the ultimate bearing capacity of the pile group foundation is enhanced.

**Fig 15:**
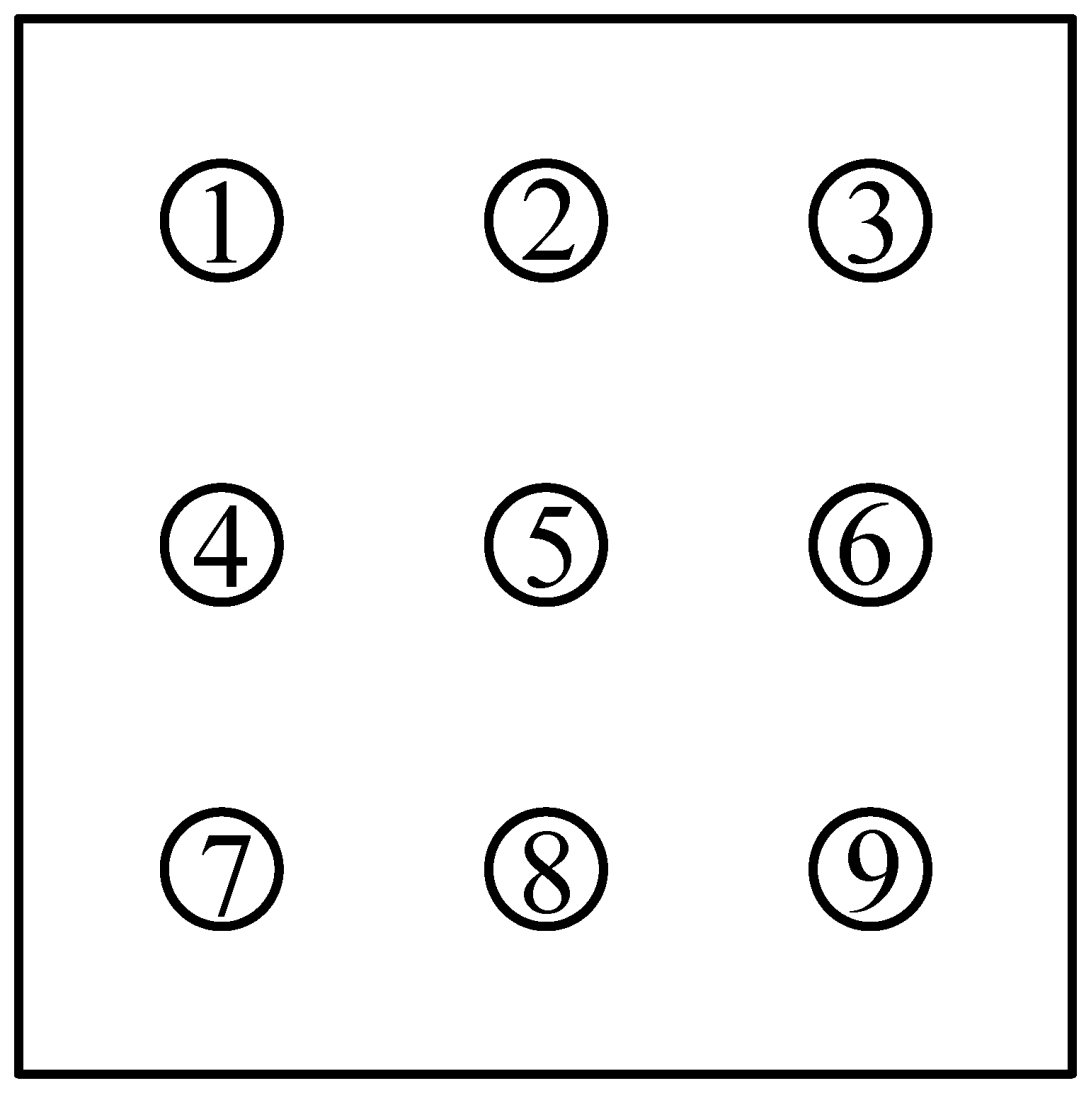
Pile number arrangement diagram.

To facilitate analysis of the mechanical behavior of individual piles within the group, each pile in the pile group foundation was assigned a numerical label as shown in Fig 15.

**Fig 16:**
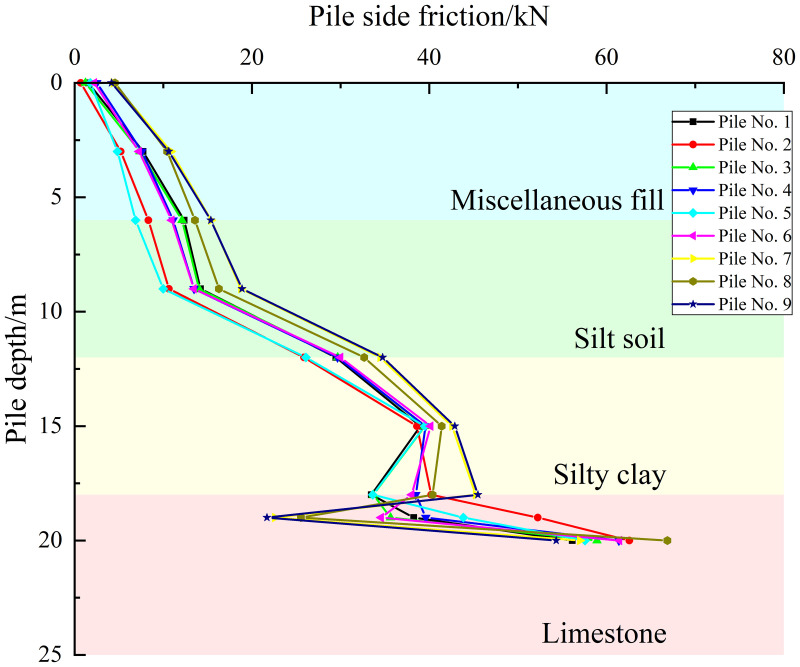
Pile side friction curve under working condition I.

**Fig 17:**
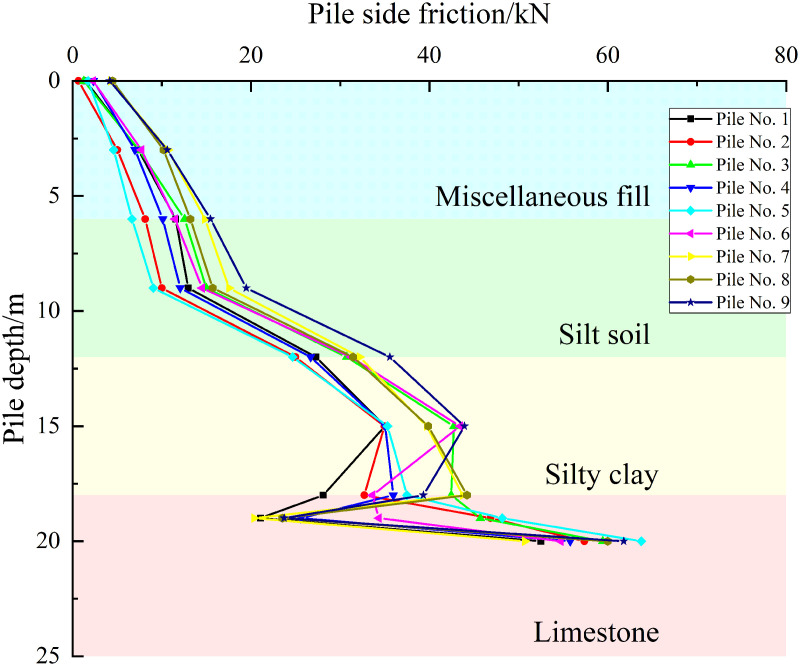
Pile side friction curve under working condition II.

**Fig 18:**
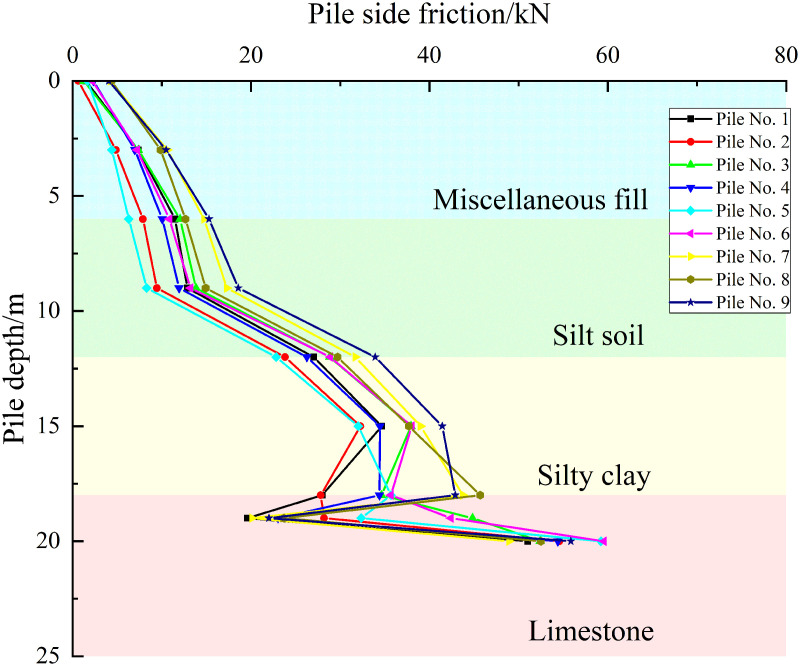
Pile side friction curve under working condition III.

Taking a uniformly distributed load of 600 kPa on the cap as an example, as shown in Figs 16–18, the pile side friction resistance curves for the three cases indicate that the primary distribution zones of side friction are the silty clay and limestone layers. However, the friction in the fill and silty soil layers is low and increases gradually. In contrast, the side friction rises rapidly in the silty clay section, marked by a sharp change in slope, and the curve experiences a sudden shift at the interface between the silty clay and limestone due to differences in mechanical properties. In the embedded rock section at the pile tip, the side resistance slows down and decreases slightly, reflecting an increased contribution from end bearing and a partial release of side resistance. The pile group effect causes similar responses in the shallow piles, while the deeper piles’ responses gradually diverge, revealing load redistribution and shielding effects due to pile-soil interaction.

For Case I, the envelope for the 12–18 m segment is the narrowest and most uniform and load transfer is approximately axisymmetric. For Case II, the scatter of the 12–18 m segment increases and piles nearer the karst cavity show a higher peak of side friction at about 16–18 m with larger differences between piles. For Case III, the scatter increases further, the peak side friction for piles close to the cavity shifts upward and increases while that for piles farther away decreases which reflects stress redistribution induced by the cavity offset and leads to stress reallocation within the pile group, some piles present a peak fall trajectory at about 16–18 m which suggests possible local sliding or softening at the interface, and overall with a constant cavity roof thickness an increase in cavity offset causes the side friction in the pile group to evolve from a uniform distribution to clear differentiation between piles.

**Fig 19:**
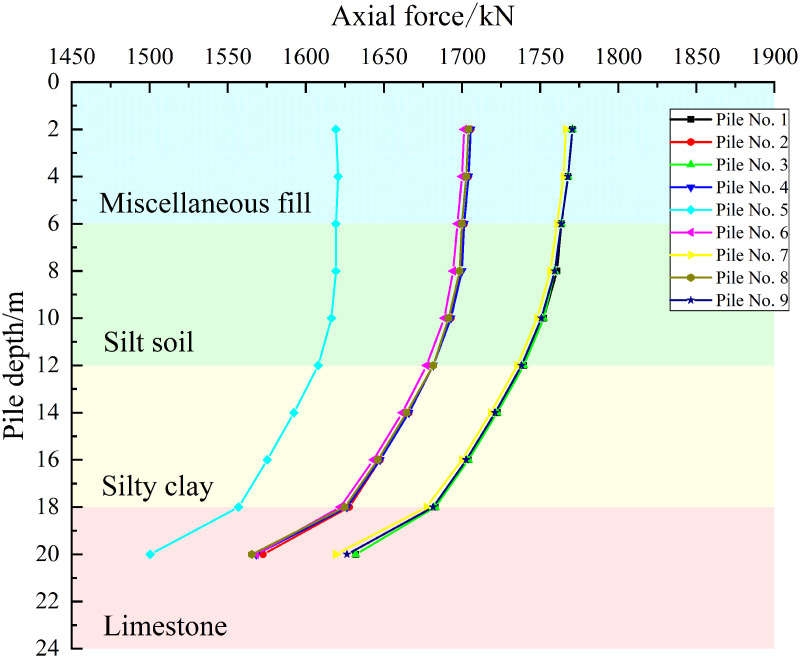
Axial force curve under working condition I.

**Fig 20:**
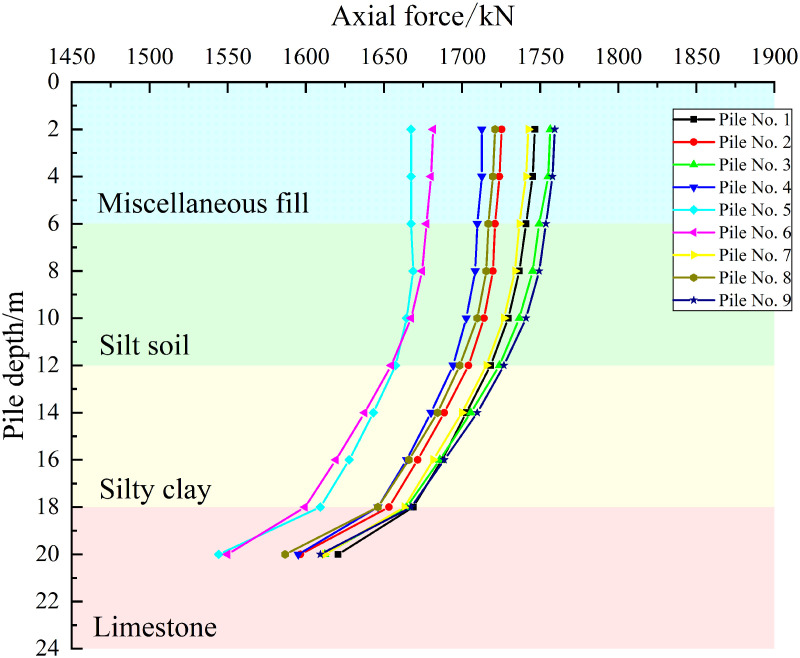
Axial force curve under working condition II.

**Fig 21:**
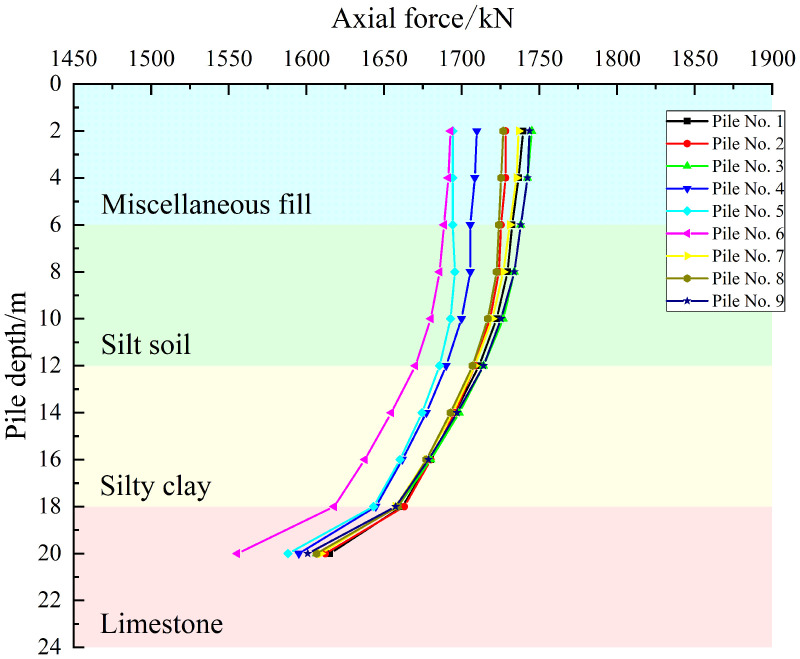
Axial force curve under working condition III.

As shown in Figs 19–21, the axial force curves of the piles under the three cases are observed to exhibit a consistent pattern in which axial force is decreased with increasing pile depth. Attenuation is gradual in the 0–12 m soil layer, while the rate of decay increases in the 12–18 m layer. A distinct inflection point is observed at the 18 m rock-soil interface, with the greatest attenuation occurring in the 18–20 m embedded rock section, where significant load loss is caused by the differences in geotechnical properties of the limestone layer.

For Case I, similar curve shapes are exhibited by the piles at the same locations in the pile group foundation, with small differences in axial force being shown at the pile tips and larger differences being observed between locations. When considered together with the layout shown in Fig 15, this indicates that the load distribution is approximately axisymmetric. For Case II, increased scatter of the axial force curves is shown for piles at the same locations, while decreased differences are observed between different locations. For Case III, markedly increased scatter is demonstrated for piles at the same locations, while further decreased differences are seen between locations. Overall, significant redistribution of load transfer in the horizontal plane within the pile group foundation is produced by increasing the offset of the karst cavity, which reduces the location-driven differences in axial force caused by the pile group effect. Under these geotechnical conditions, the bearing capacity of the pile group foundation is provided primarily by pile tip resistance.

**Fig 22:**
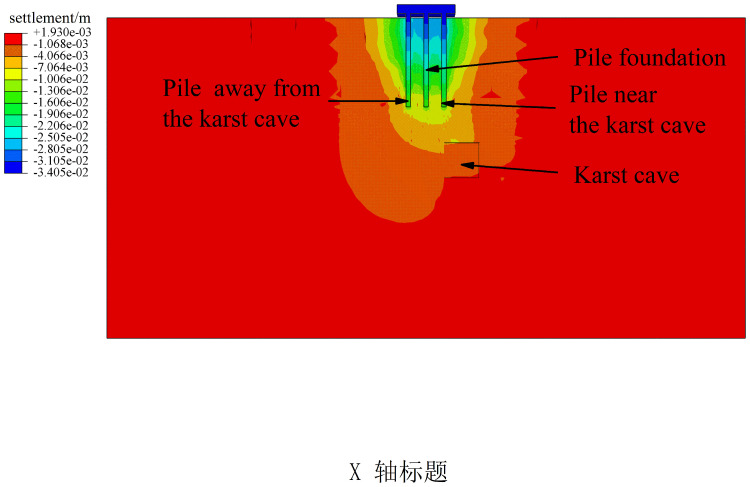
Displacement cloud map of pile group condition III under uniformly distributed load of 600 kPa.

Based on the side friction resistance curve and axial force distribution curve of the pile group foundation, it can be concluded that the pile group foundation used in this study behaves overall as a friction-end bearing pile. Studies [29,52,53] indicated that piles in different positions within the pile group foundation share different loads. Corner piles bear the largest load, followed by edge piles, while middle piles bear the least. For piles at the same location within the pile group foundation, the side friction resistance of piles closer to the karst cave is higher than that of piles farther from the cavity. This is because the full exertion of side friction resistance depends on the relative displacement between the pile and the soil. Specifically, the greater the pile-soil relative displacement, the more fully the side friction resistance is developed. In the presence of underlying karst caves, the overall stiffness and constraint ability of the overlying rock layer are reduced, making local areas more prone to concentrated deformation. Moreover, study [30] showed that under the same load conditions, the closer a single pile in a karst area is to the cave, the more fully its side friction resistance is developed. A pile group foundation consists of multiple single piles and a pile cap, and the characteristics of side friction resistance development are somewhat similar to those of single piles. As shown in Fig 22, under condition III, the presence of a karst cave causes significant spatial non-uniformity in soil deformation beneath the pile group foundation, with soil displacements closer to the cave larger than those farther away. However, the displacements of individual piles within the pile group foundation are constrained by the pile cap, resulting in relatively small differences in individual pile displacements. It causes greater pile-soil relative displacement for piles near the cave, allowing their side friction resistance to be more fully realized. From Fig 10(c), it can be seen that the side friction resistance of corner piles No. 3 and No. 9 near the cave is greater than that of corner piles No. 1 and No. 7 farther from the cave. Meanwhile, since the bearing capacity of the pile foundation is shared between pile-end resistance and side friction resistance, when the side friction resistance of piles near the cavity increases, their pile-end resistance correspondingly decreases, exhibiting a certain degree of ‘end resistance unloading’ effect. Piles farther from the cave experience higher stiffness from the surrounding rock and soil, less overall deformation, and limited pile-soil relative displacement, resulting in less fully developed side friction resistance and relatively lower side friction resistance values.

### 4.6 Verification of the rationality of the ultimate bearing capacity calculation equation

**Fig 23:**
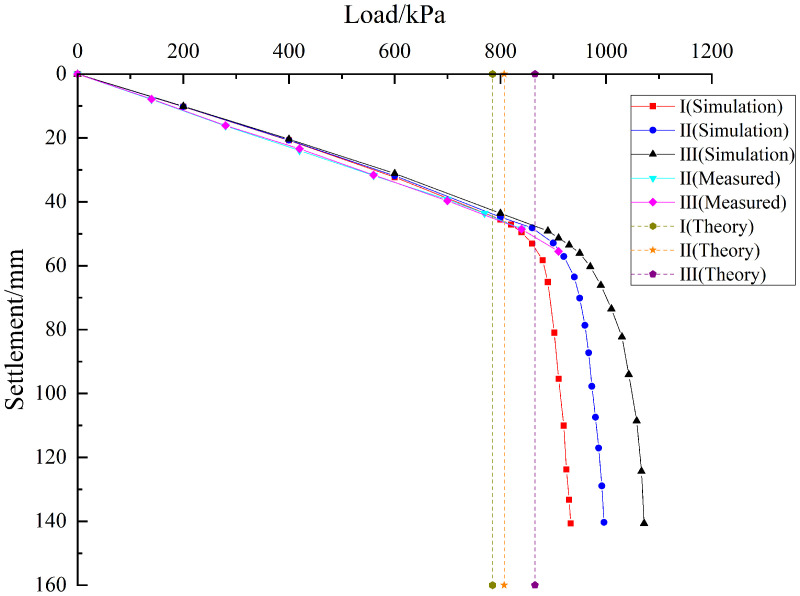
Comparison of ultimate bearing capacity of pile groups under the condition of underlying offset cave.

Based on pile foundation engineering in the karst area of the Baiyun District Science and Technology Manufacturing Park in Guangzhou, Guangdong Province, a rationality verification analysis was performed for the calculation equation of ultimate bearing capacity of pile group foundations under conditions involving underlying karst cave displacement proposed in this paper. By substituting the mechanical parameters of the soil and rock listed in Table 7 into Equations (2), (23), (27), and (29), the theoretical ultimate bearing capacities corresponding to conditions I, II, and III are calculated as 785.36 kPa, 807.26 kPa, and 865.39 kPa, respectively. As shown in Fig 23, the calculation method proposed in this paper is evaluated through a comparison based on theoretical analysis, field measurement, and numerical simulation. From the above analysis, the numerical simulation result indicates that the ultimate bearing capacity of the pile group foundation under condition I is approximately 825 kPa. Under condition II, the numerical simulation result indicates that the ultimate bearing capacity of the pile group foundation is approximately 850 kPa. The field monitoring result is approximately 770 kPa. Under load condition III, the numerical simulation result indicates that the ultimate bearing capacity of the pile group foundation is approximately 900 kPa. The field monitoring result is approximately 840 kPa. The analysis indicates that under load condition I, the error between the theoretical calculation and the numerical simulation result is approximately 5.05%. Under load condition II, the error between the theoretical calculation and the numerical simulation result is approximately 5.29%. The error between the theoretical calculation and the field monitoring result is approximately 4.84%. Under load condition III, the error between the theoretical calculation and the numerical simulation result is approximately 3.99%. The error between the theoretical calculation and the field monitoring result is approximately 3.02%. The analysis indicates that the errors fall within a reasonable range. The theoretical calculation method proposed in this paper is therefore considered rational.

## 5 Discussion

### 5.1 Effect of karst cave to pile tip distance on the bearing capacity of pile group foundations

**Fig 24:**
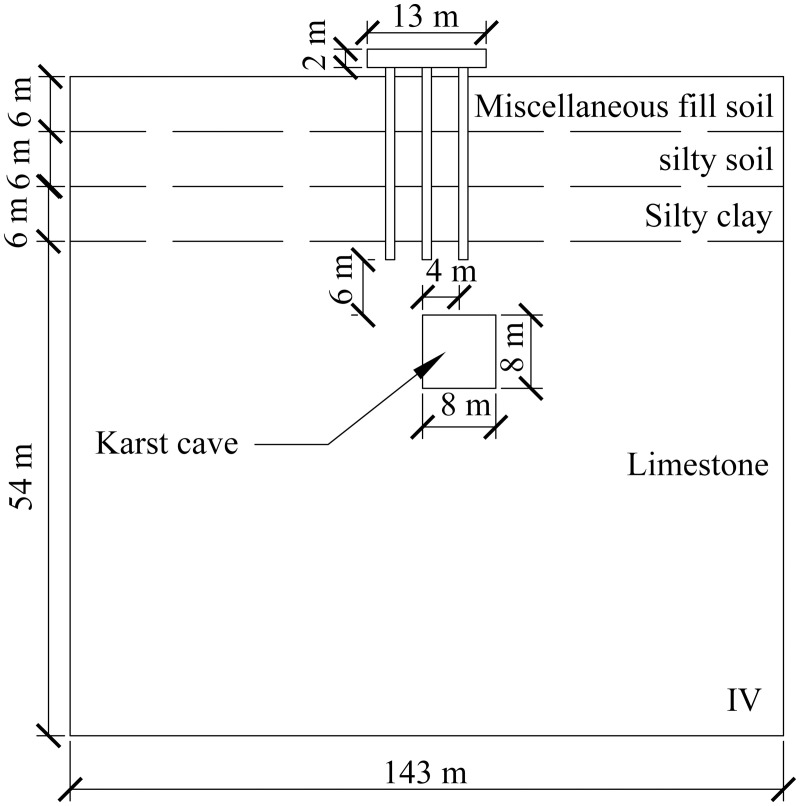
The cave with a 6 m offset from the pile end.

**Fig 25:**
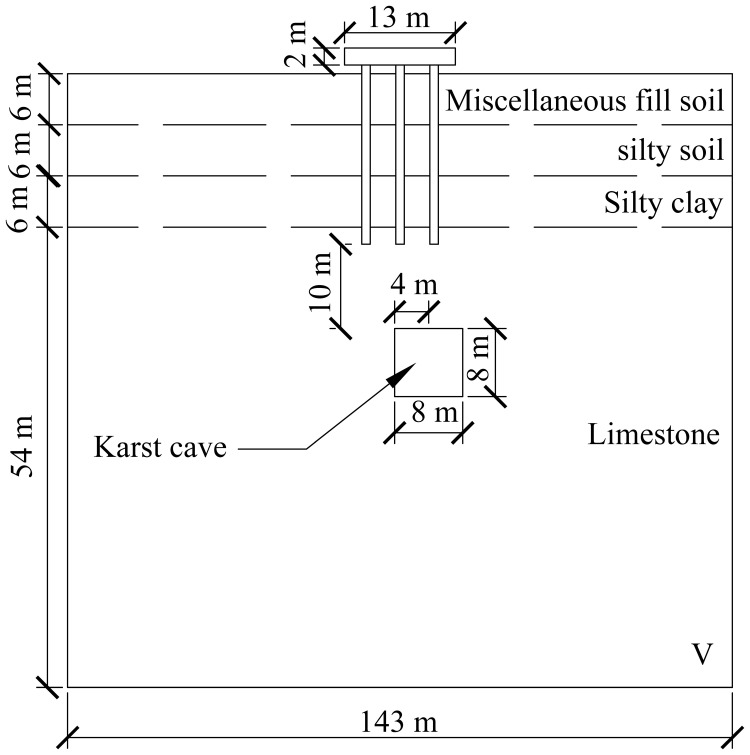
The cave with 10 m offset from the pile end.

To verify the applicability of the theoretical equation, the relationship between the ultimate bearing capacity of the pile group foundation and the height of the karst cavity above the pile tip was investigated. As shown in Figs 24, 25, the offset of the cavity below is 4 m. The case with a karst cavity located 6 m from the pile tip is defined as Condition IV, whereas the case with a distance of 10 m is defined as Condition V.

**Fig 26:**
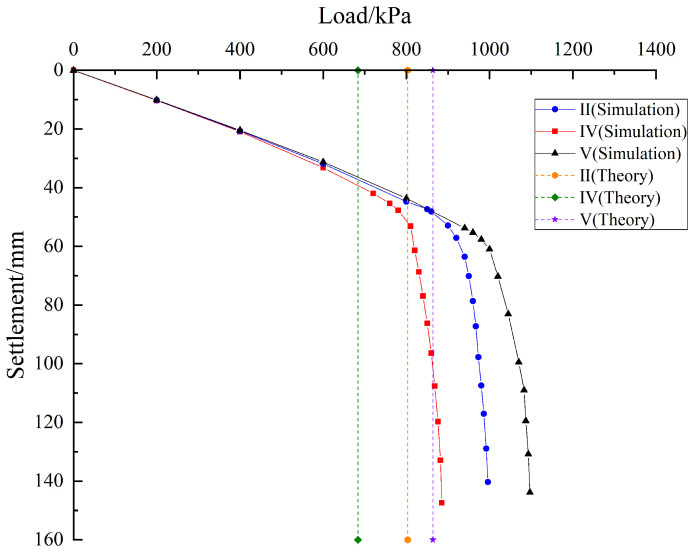
Load-settlement curves for different distances between cave and pile end.

As shown in Fig 26, the load settlement curves under the three cases are linear in the early stage and then become nonlinear with rapid settlement after the ultimate bearing capacity of the pile group foundation is reached. From the Fig, it can be observed that a distinct inflection point appears at about 720 kPa in Case IV, at about 850 kPa in Case II, and at about 940 kPa in Case V. According to the ‘Technical Specification for Testing of Building Foundation Pile’ (JGJ 106–2014), the ultimate bearing capacities are approximately 720 kPa for Case IV, 860 kPa for Case II, and 940 kPa for Case V. Substituting the above conditions and the rock and soil param in Table 7 into theoretical Equations (29) gives 683.59, 807.26, and 863.69 kPa for Cases IV, II, and V. The differences between the simulation and theoretical results are about 5.34% for Case IV, 5.29% for Case II, and 8.84% for Case V, which are within a reasonable range and further support the applicability of the proposed method. Both the numerical simulations and the theoretical analysis indicate that the ultimate bearing capacity is largest for Case V, followed by Case II, and then Case IV.

### 5.2 Reasonable selection of the group pile effect coefficient

In the process of determining the pile group effect coefficient, the stress superposition method was adopted. To verify the rationality of the selected method, working condition I was used as a representative case to compare and analyze pile group effect calculation methods. The results are presented in Table 11.

**Table 11:** Analysis of calculation methods for the pile group effect.

Name	Equation	Group pile effect coefficient	Theoretical calculation results
Entity perimeter method	$\begin{array}{c} \eta =\frac{2\left(m+n-2\right)⬝r+4D}{\pi mnD} \end{array}$	1.2732	unreasonable
Converse-Labarre method	$\begin{array}{c} \eta =1-\left({tan}^{-1}\frac{D}{r}\right)⬝\frac{2\left[\left(n-1\right)m+\left(m-1\right)n\right]}{\pi mn} \end{array}$	0.9491	872.10 kPa
Seiler-Keeney method	$\eta =\left[1-\frac{11r}{7\left(r^{\text{2}}-1\right)}⬝\frac{m+n-2}{m+n-1}\right]+\frac{0.3}{m+n}$	0.7148	656.81 kPa
Partial coefficient method	The coefficient of pile group effect is derived based on a large number of experimental results.	–	–
Stress superposition method	Equation (2–6)	0.8547	785.36 kPa

As shown in Table 11, the solid perimeter method is not suitable for the calculations presented in this paper. The Converse Labarre method produces results that are higher than the measured monitoring values. The Seiler Keeney method produces results that are significantly lower than the measured monitoring values. The partial factor method requires extensive experimental data to determine group pile effect coefficients. Compared with other methods, the stress superposition method produces calculation results that are closer to the measured values. The associated error remains within a reasonable range. The stress superposition method is therefore selected to evaluate the group pile effect in this paper.

### 5.3 Sensitivity analysis of parameters

Studies [54–57] have shown that factors such as the geometric characteristics of karst caves, the arrangement parameters of pile groups, and the mechanical properties of the rock layer at the pile ends all affect the development of the end-bearing resistance of group pile foundations. Therefore, we focus on analyzing the effect of parameter factors on the pile end resistance *Q*_*P*_. For Equation (23), the main calculation parameters of *Q*_*P*_ are the width of the pile end stress concentration zone *b*, the cave offset *s*, the thickness of the cave roof *h*, the internal friction angle *φ* of the rock, and the cohesion *c* of the rock mass. To avoid scale effects, we set the analytical factors as *b*, *h*/*b*, *s*/*b*, *φ*, and *c*.

Based on the theoretical sensitivity analysis method [58,59], bearing capacity function models of the form *Q*_*P*_ = *z*(*xi*) are established for *b*, *h*/*b*, *s*/*b*, *φ*, and *c*, as shown in Equation (30). The corresponding sensitivity function y(xi) is obtained from the derivative of this function, as shown in Equation (31).


$$Q_{P}=f(x_{\text{1}},x_{\text{2}}......x_{n})=y\left(x\right)$$ (30)

$$z\left(x\right)=\left|\frac{dy\left(x\right)}{dx}\right|⬝\frac{x}{y\left(x\right)}$$ (31)

where *Q*_*P*_ represents the pile end resistance, *x* denotes the primary influencing parameter, and *z(xᵢ)* represents the sensitivity function.

Condition II was adopted as the baseline model to evaluate the pile end resistance for each parameter variable. The baseline parameter values are listed in Table 12.

**Table 12:** Reference parameter table.

*b*	*h/b*	*s/b*	*φ*	*c*
8	1	0.5	34°	6 Mpa

Taking Condition II as an example, the optimal solutions are *θ*_1_ = 12.1°, *θ*_2_ = 27.3°, *θ*_3_ = 43.6°, and *θ*_4_ = 66.8°. By substituting these values into Equation (23), *Q*_*P*_ = 782.43 kPa. Similarly, each parameter condition is solved sequentially. The parameter variables and corresponding *Q*_*P*_ values are presented in Table 13.

**Table 13:** Parameter variables and pile end resistance *Q*_*P*_*.*

*b*	*Q*_*P*_	*h/b*	*Q*_*P*_	*s/b*	*Q*_*P*_	*φ*	*Q*_*P*_	*c*	*Q*_*P*_
8	782.43	0.5	567.27	0.5	782.43	24°	663.72	4 MPa	521.64
8.5	769.24	0.75	658.73	1.0	825.39	29°	718.95	5 MPa	652.35
9	742.65	1	782.43	1.5	865.27	34°	782.43	6 MPa	782.43
9.5	716.93	1.25	838.83	2.0	900.61	39°	856.88	7 MPa	912.75
10	687.32	1.5	880.56	2.5	930.83	44°	943.24	8 MPa	1043.22
10.5	662.65	1.75	937.62	3.0	955.94	49°	1061.56	9 MPa	1173.69

**Fig 27:**
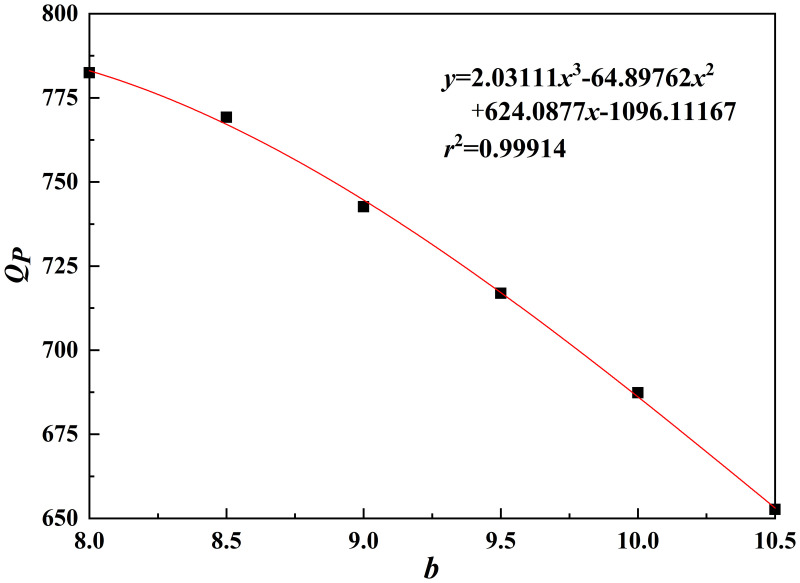
*b*-*Q_P_* fitting plot.

**Fig 28:**
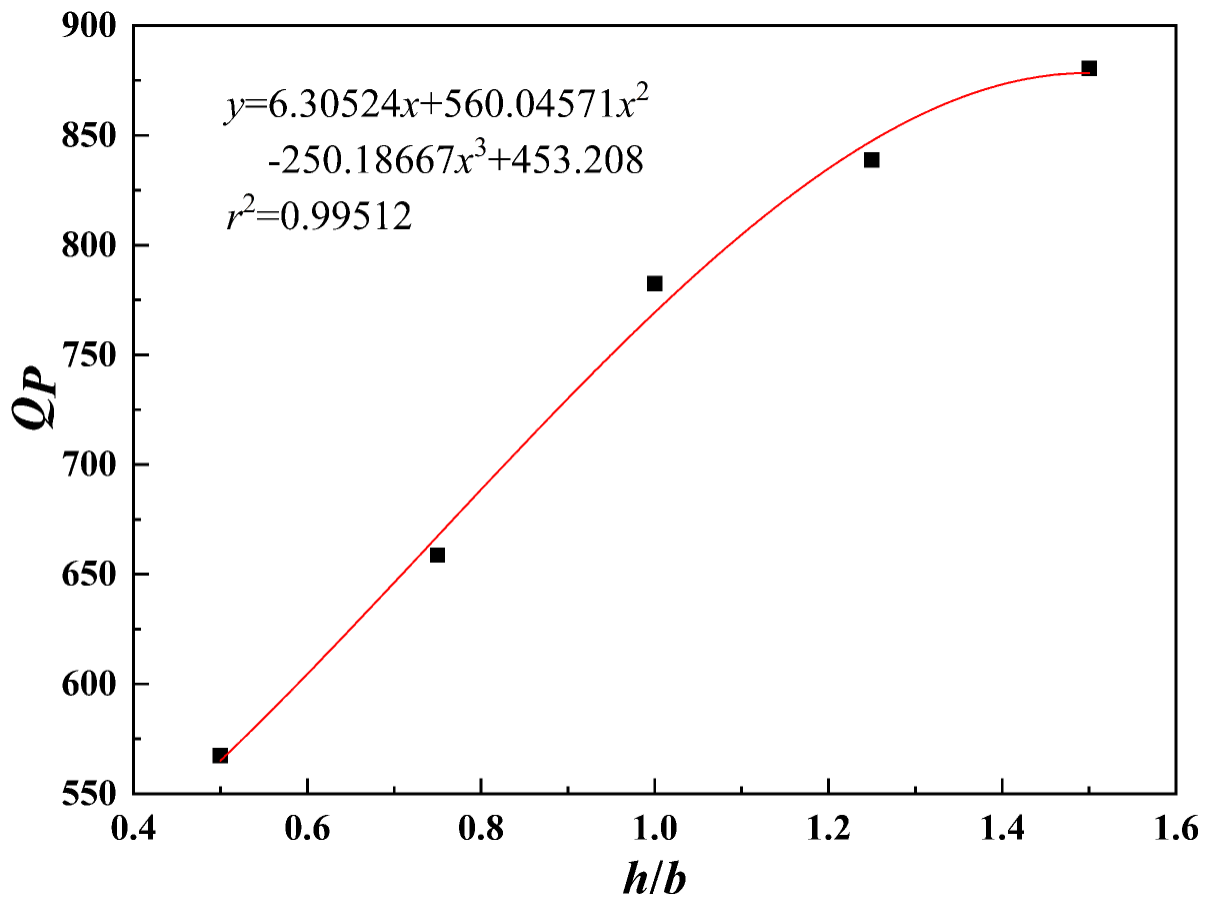
*h*/*b*-*Q_P_* fitting plot.

**Fig 29:**
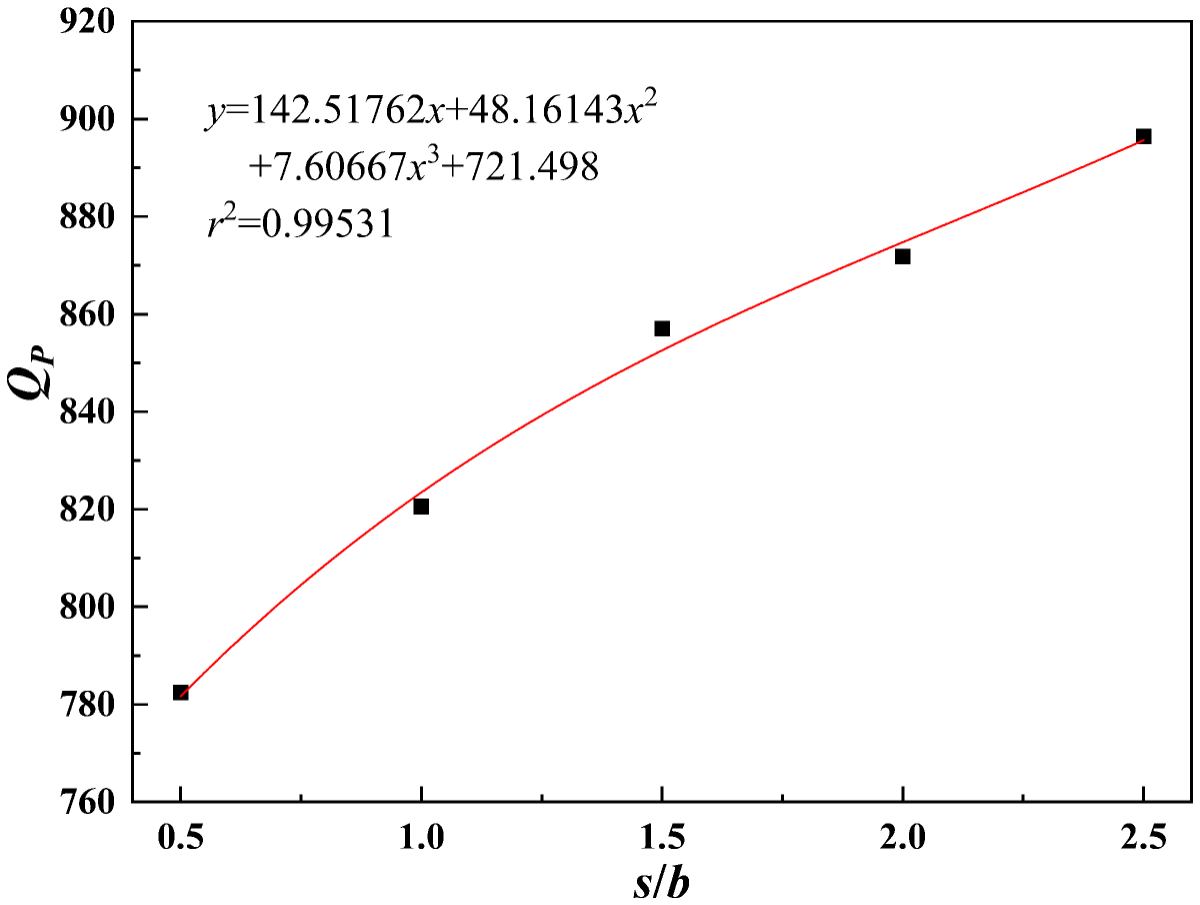
*s*/*b*-*Q_P_* fitting plot.

**Fig 30:**
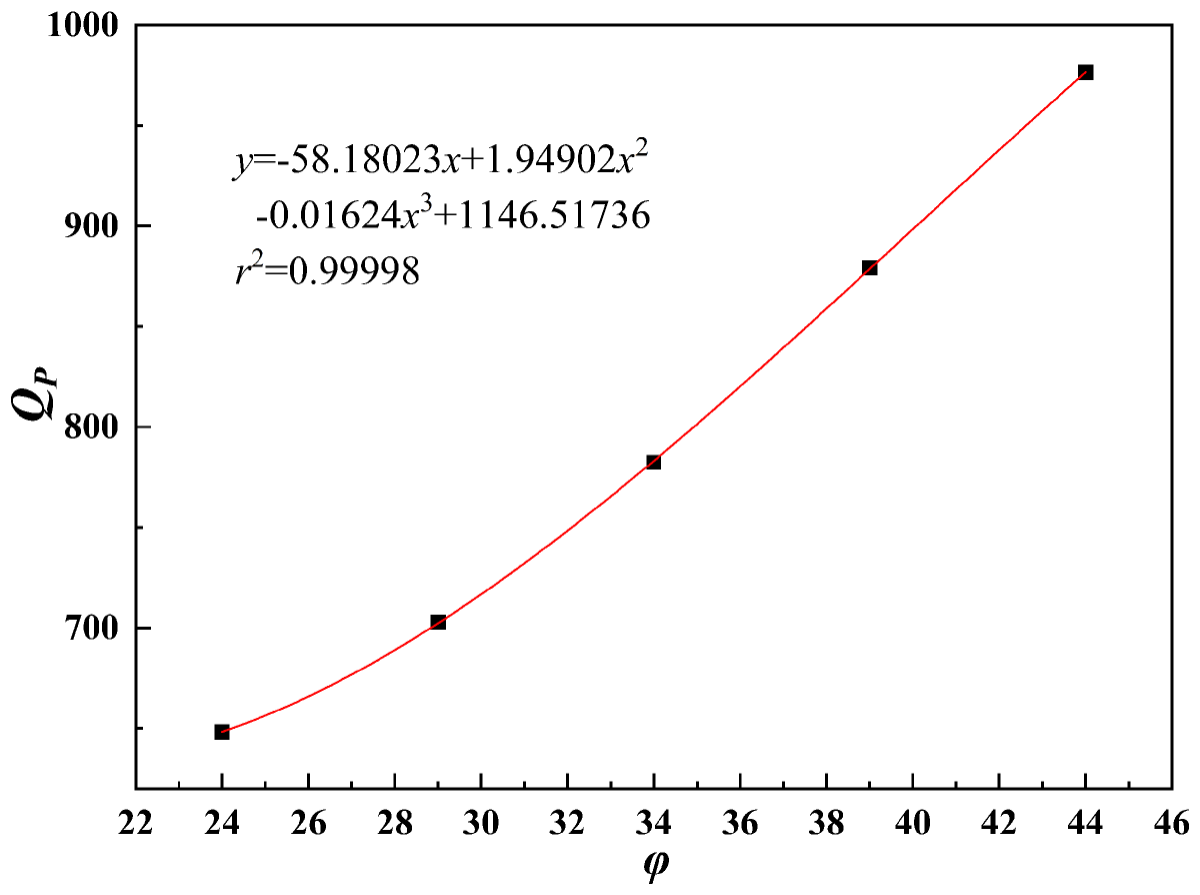
*φ*-*Q_P_* fitting plot.

**Fig 31:**
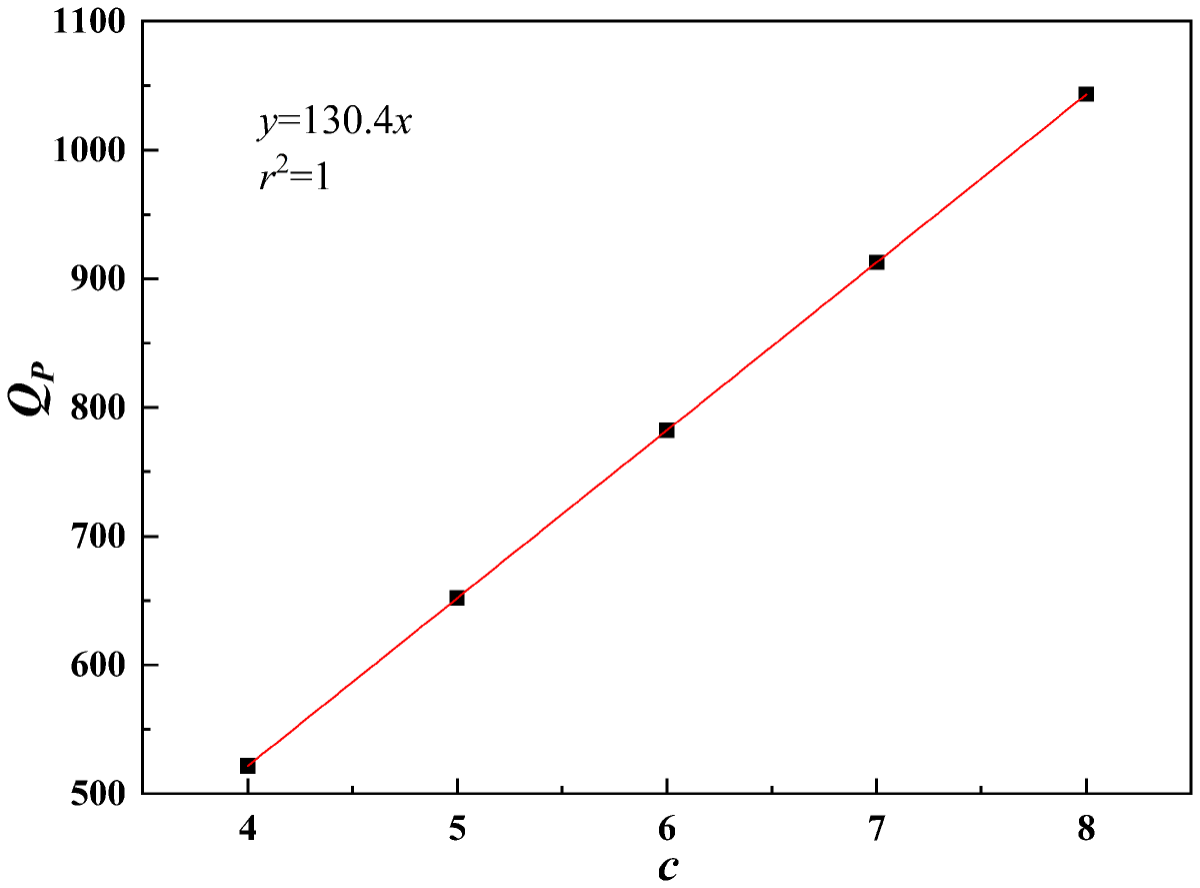
*c*-*Q_P_* fitting plot.

As shown in Figs 27–31, the fitting accuracy of each parameter is high by fitting the calculation results, which indicating that the established functional relationship can reflect the law of *Q*_*P*_ changing with the parameters.

The sensitivity function is obtained according to Equation (31). The sensitivity factor is calculated by substituting the baseline parameters into the sensitivity function. The results are presented in Table 14.

**Table 14:** Sensitivity function and sensitivity factor.

*x*	Sensitivity functions	Sensitivity factors
*b*	$\begin{array}{c} z\left(x\right)=\frac{x\left|6.09333x^{\text{2}}-129.79524x+624.0877\right|}{2.03111x^{\text{3}}-64.89762x^{\text{2}}+624.08771x-1096.11167} \end{array}$	0.2483
*h*/*b*	$\begin{array}{c} z\left(x\right)=\frac{x⬝\left|130.38222x^{\text{2}}-579.00572x+766.77942\right|}{\text{43.46074}x^{\text{3}}\text{-289.50286}x^{\text{2}}\text{+766.77942}x\text{+245.50333}} \end{array}$	0.4152
*s*/*b*	$\begin{array}{c} z\left(x\right)=\frac{x⬝\left|30.98889x^{\text{2}}-157.5254x+221.8791\;\right|}{\begin{array}{c} \text{10.32963}x^{\text{3}}\text{-78.7627}x^{\text{2}}\text{+221.8791}x\text{+690.38667} \end{array}} \end{array}$	0.0964
*φ*	$\begin{array}{c} z\left(x\right)=\frac{x⬝\left|0.00918x^{\text{2}}-0.08972x+6.3919\right|}{\begin{array}{c} \text{0.00306}x^{\text{3}}\text{-0.04486}x^{\text{2}}\text{+6.3919}x\text{+484.05078} \end{array}} \end{array}$	0.6163
*c*	$\begin{array}{c} z\left(x\right)=\frac{x⬝\left|130.37657\right|}{\begin{array}{c} \text{130.37657}x\text{+0.23229} \end{array}} \end{array}$	0.9997

The results indicate that the sensitivity factor for cohesion *c* is approximately 1, because *Q*_*P*_ varies linearly with cohesion *c* in Equation (23), resulting in a strong effect. The sensitivity factor for the internal friction angle *φ* is 0.6163, indicating that it is the second most influential parameter. An increase in the internal friction angle *φ* enhances soil shear strength and improves the stability of the pile–soil system under external loading. The ratio of top plate thickness to pile group length *h*/*b* is 0.4152. Although variations in this ratio affect the stiffness and failure mode of the overlying bearing structure, the impact is limited under the selected reference conditions. The sensitivity factor associated with the stress concentration zone width *b* is 0.2483, which is significantly lower than those of cohesion *c*, internal friction angle *φ*, and the ratio *h*/*b*. Within the selected parameter range, *b* has a relatively weak effect on ultimate bearing capacity. This is because, under the adopted upper-bound limit analysis framework, *b* affects bearing capacity indirectly by modifying the geometric scale and location of the failure mechanism. Moreover, because the failure mode is primarily governed by cavity geometry and top plate conditions, variations in *b* within a reasonable range do not alter the fundamental form of the optimal failure mechanism and only produce secondary adjustments in bearing capacity. Therefore, *b* is taken as the width of the equivalent stress concentration zone for modelling simplification. The ratio of karst cavity offset distance to pile group length *s*/*b* is 0.0964, indicating that cavity offset has only a minor effect on pile end resistance *Q*_*P*_. This is consistent with the mechanism whereby karst cavity offset mainly influences the failure mode and propagation range of the underlying bedrock.

### 5.4 Limitations of the study

In studies of pile foundation bearing capacity and settlement in karst areas, the geometry of karst cavities is typically idealized to ensure the feasibility of theoretical and numerical analyses. Previous studies often simplify karst cavities as circular or rectangular shapes to establish mechanical models and conduct parametric analyses. This simplification reduces computational instability caused by irregular geometries and imposes clearer geometric constraints on stress distribution and failure zones, thereby improving result consistency and comparability. Studies [60,61] suggest that rectangular karst cavity models better represent the effects of real cavities on pile foundation bearing behavior and also improve mesh convergence in finite element modeling. Therefore, rectangular karst cavity models are adopted in the numerical analysis.

However, actual karst cavities often exhibit irregular geometries and stress responses associated with arching effects and spatial stress redistribution mechanisms. In contrast, the roof slab of a rectangular karst cavity is planar and lacks the stress dispersion effect induced by curvature. Under the same span conditions, a bending-controlled stress state develops more readily, and local tensile stresses are relatively high. Consequently, under equivalent geometric dimensions, rectangular karst cavity models tend to produce unfavorable roof slab stress and deformation responses, and the predicted bearing capacity and settlement are generally conservative. From an engineering perspective, this assumption supports conservative safety estimates but provides only a limited representation of the complex geometry of real cavities. Future work will incorporate measured cavity morphology data to perform numerical analyses under irregular karst conditions, thereby improving model representativeness.

In numerical simulations, the Mohr–Coulomb constitutive model is used to describe the mechanical behavior of soil and rock masses. Based on the shear yield criterion, the model characterizes material strength using the internal friction angle and cohesion. The model is concise, and its parameters have clear physical meaning, which makes it widely applicable in geotechnical numerical analysis. Studies [62,63] indicate that the Mohr–Coulomb model is well suited to describing overall yielding and plastic zone development in soil and rock masses dominated by shear failure, and is particularly appropriate for macroscopic analyses of bearing capacity and stability. However, the model does not explicitly account for crack propagation, tensile failure, or progressive damage evolution, and therefore has limitations in representing brittle failure and tensile softening in rock. This study investigates the overall bearing capacity and failure response of a pile group foundation–limestone system under overburden loading. Numerical results and plastic zone distributions show that rock mass failure is dominated by shear plastic development and shear strain concentration near the pile tip and cavity roof. The overall instability mode is primarily governed by shear deformation, while the tensile stress control zone is limited. Under these stress conditions, the Mohr-Coulomb model represents the rock mass as an equivalent continuum and can capture general stress redistribution trends and ultimate bearing capacity response.

However, the model does not include a separate tensile failure criterion or damage evolution parameters, which increases the equivalent tensile and residual bearing capacity and leads to more conservative predictions. For failure scenarios governed by tensile crack propagation and block instability, this simplification may underestimate the extent of local failure. Future research could integrate experimental data and introduce the Hoek–Brown strength criterion or a continuous damage model to describe nonlinear strength and brittle failure in rock masses, thereby improving the accuracy and applicability of numerical analyses.

The soil layer is assumed to be homogeneous in this study. However, this assumption may not fully reflect field conditions. Future research could adopt spatially variable random field models [64] or the Burgers constitutive model [65] to better simulate the effects of complex soil stratification on pile foundations and improve model accuracy and applicability. In addition, the effects of dynamic loading and long-term creep behaviour were not considered. Future studies should further investigate the contributions of dynamic loading, seismic actions, and soil creep to pile foundation settlement and stability. By considering these factors, the performance and long-term stability of pile group foundations in karst areas can be more comprehensively evaluated, providing a more scientific theoretical basis for engineering design.

## 6 Conclusion

Based on the pile foundation engineering at the Guangzhou Baiyun District Science and Technology Manufacturing Park, this study analyses the bearing capacity and bearing characteristics of a pile group foundation in the presence of underlying offset karst cavities through theoretical analysis, field monitoring and numerical simulation. The principal conclusions are as follows.

(a) This study derives an analytical equation for the ultimate bearing capacity of pile group foundations above an underlying offset cavity by combining pile group effect theory with a solution for shaft friction and the upper bound theory of limit analysis for tip resistance. Based on the Science and Technology Manufacturing Park project in Baiyun District, the model was validated against monitoring and numerical simulation. The errors between theoretical and simulated capacities are about 5.05% for Case I, 5.29% for Case II, 3.99% for Case III, 5.34% for Case IV, and 8.84% for Case V. The error between theoretical and field results is about 4.84% for Case II and 3.02% for Case III.
(b) The results indicate that a larger lateral offset of the underlying cavity increases the ultimate bearing capacity of the pile group foundation, with the capacities ranked as Ⅲ > Ⅱ > Ⅰ. Similarly, a higher cavity roof above the pile toes results in a higher ultimate capacity, with the ranking of Ⅴ > Ⅱ > Ⅳ. These trends suggest that the design and layout should maximize the distance between piles and cavities to enhance the ultimate bearing capacity of the pile group foundation.

### List of symbols

**Table:** 

W_U_	Ultimate bearing capacity of pile group foundation
P_U_	Ultimate bearing capacity of single pile
a	Number of piles in a pile group foundation
η	Pile group effect coefficient
λ	Average reduction factor of pile group foundation
m	Number of transverse pile foundations in pile group system
n	Number of longitudinal pile foundations in pile group system
φ	Internal friction angle of soil layer
l	Pile length
r_1_	Lateral pile spacing
r_2_	Longitudinal pile spacing
d	Cave width
b	The width of the pile tip influence zone of a pile group foundation.
h	Bedrock thickness
s	Cave offset
e	Natural base
E	Elastic modulus
ω	Angular velocity
γ_i_	Specific gravity of rock and soil layer
z	Depth of pile in rock and soil
k_i_	Friction coefficient of corresponding rock and soil layer
ρ	Density
c	Cohesion
v	Poisson’s ratio
φ	Internal friction

### Methods

The data used in this study were obtained from static load tests on pile foundations, carried out by the research team at the Bai-Yun District Science and Technology Manufacturing Park in Guangzhou. These tests formed part of the routine construction process and were authorized by the project owner for scientific research purposes. The study did not involve experiments in protected areas, humans, or animals and therefore required no additional permits.

## Supporting information

S1 Data Minimal dataset including raw values underlying all Figs and tables. (ZIP)
